# Bayesian phylodynamics of avian influenza A virus H9N2 in Asia with time-dependent predictors of migration

**DOI:** 10.1371/journal.pcbi.1007189

**Published:** 2019-08-06

**Authors:** Jing Yang, Nicola F. Müller, Remco Bouckaert, Bing Xu, Alexei J. Drummond

**Affiliations:** 1 College of Global Change and Earth System Science, Beijing Normal University, Beijing, China; 2 School of Computer Science, University of Auckland, Auckland, New Zealand; 3 Centre for Computational Evolution, University of Auckland, Auckland, New Zealand; 4 Department of Biosystems Science and Engineering, ETH Zürich, Zürich, Switzerland; 5 Swiss Institute of Bioinformatics (SIB), Lausanne, Switzerland; 6 Max Planck Institute for the Science of Human History, Jena, Germany; 7 Department of Earth System Science, Tsinghua University, Beijing, China; University of Chicago, UNITED STATES

## Abstract

Model-based phylodynamic approaches recently employed generalized linear models (GLMs) to uncover potential predictors of viral spread. Very recently some of these models have allowed both the predictors and their coefficients to be time-dependent. However, these studies mainly focused on predictors that are assumed to be constant through time. Here we inferred the phylodynamics of avian influenza A virus H9N2 isolated in 12 Asian countries and regions under both discrete trait analysis (DTA) and structured coalescent (MASCOT) approaches. Using MASCOT we applied a new time-dependent GLM to uncover the underlying factors behind H9N2 spread. We curated a rich set of time-series predictors including annual international live poultry trade and national poultry production figures. This time-dependent phylodynamic prediction model was compared to commonly employed time-independent alternatives. Additionally the time-dependent MASCOT model allowed for the estimation of viral effective sub-population sizes and their changes through time, and these effective population dynamics within each country were predicted by a GLM. International annual poultry trade is a strongly supported predictor of virus migration rates. There was also strong support for geographic proximity as a predictor of migration rate in all GLMs investigated. In time-dependent MASCOT models, national poultry production was also identified as a predictor of virus genetic diversity through time and this signal was obvious in mainland China. Our application of a recently introduced time-dependent GLM predictors integrated rich time-series data in Bayesian phylodynamic prediction. We demonstrated the contribution of poultry trade and geographic proximity (potentially unheralded wild bird movements) to avian influenza spread in Asia. To gain a better understanding of the drivers of H9N2 spread, we suggest increased surveillance of the H9N2 virus in countries that are currently under-sampled as well as in wild bird populations in the most affected countries.

## Author summary

What drives the geographic dispersal and genetic diversity of avian influenza A virus H9N2 in Asia? We used two model-based approaches, DTA and MASCOT, to reconstruct the phylogeographic dynamics of the virus genetic sequence and predictor data. To do so, we compiled multiple potential predictors to inform migration rates and effective population size in a generalized linear model framework. This allowed us to quantify which of these predictors most likely predicts the spread of avian influenza A/H9N2 in Asia. We found a positive association of international poultry trade and national poultry production time-series with virus migration rates and effective population sizes respectively. We also identified geographic proximity as a strongly supported driver to virus migration rates and this points to the potential role of wild bird populations in virus dispersal across countries. Our study is a practical example of the use of temporal information in predictors to model heterogeneous spatial diffusion and population dynamic processes and provides direction to H9N2 control efforts in Asia.

## Introduction

### Phylogeographic models and their extended GLMs

Phylogeographic methods can infer the migration history of sampled lineages based on genetic data. The discrete trait analysis (DTA) and structured coalescent model are commonly used probabilistic model-based phylogeographic methods. The DTA model treats the migration of lineages between different geographic locations as a per-lineage continuous-time Markov process, analogous to the DNA substitution process [1]. This approach achieves computational efficiency by integrating over all possible migration histories using the efficient tree pruning algorithm for computing phylogenetic likelihoods [2]. One drawback of this approach is the assumption of independence of the tree generating process and the migration process, which can lead to underuse of the data [3]. Another drawback is the potential biases in migration rates estimates when sampling is biased across sub-populations, since such a model assumes that the sample sizes across sub-populations are proportional to the subpopulation sizes (sub-populations refer to different geographic locations in this study) [3].

The structured coalescent on the other explicitly models how lineages coalesce within and migrate between sub-populations [4]. Additionally, the structured coalescent conditions on sampling and only assumes that the samples are drawn at random from a large population. This makes the structured coalescent more robust to sampling bias [3, 6]. Exact inference under the structured coalescent is challenging [5]. Thus, approximations to the structured coalescent model by approximately integrating over all ancestral migration histories were proposed [3, 6]. The marginal approximation of the structured coalescent (MASCOT) currently provides the closest approximation to the structured coalescent while being computationally efficient. This allows to analyse datasets with many different sub-populations [6, 7] and currently enables to analyse datasets of up to 500 sequences and up to 14 different states [8].

The generalized linear models (GLMs) can be employed as an extension of phylogeographic inference to inform the pathogen migration rates between distinct geographical locations by predictor data [9]. Some authors used GLMs in both discrete and continuous phylogeographic models to investigate the impact of underlying environmental variables on the dispersal frequencies and velocity of a virus respectively [10]. But only univariate models can be considered in the current GLM implementation in continuous phylogeographic inference. The GLM model incorporating multiple predictors in DTA [9] gained popularity for its computational efficiency and user-friendly implementation in BEAST 1.10 [11]. Recently, DTA GLM models were also applied to inform the potential factors shaping the spatial dispersal of influenza A virus [9, 12], the Ebola virus [13], the Foot-and-Mouth disease virus [14] and dengue virus [15], and the underlying predictors contributing to host transmission dynamics of rabies virus [16]. But it unrealistically assumes time-homogeneous substitution processes between sub-populations. The epoch GLM has already allowed for time-dependence in both coefficients and predictors data to model the heterogeneous spatial diffusion processes through time in DTA [13, 17]. However, a recent phylodynamic GLM only considered time-dependent coefficients (rather than time-dependent predictors) to inform the temporal dynamics in the spread of Ebola virus [13]. Very recently the GLM model with both time-dependent predictors and coefficients were proposed in MASCOT [8]. This allows, for the first time, the ability to quantify the contribution of both time-series and constant predictors to both migration rates and effective population sizes jointly in a structured population.

### Avian influenza A virus H9N2 in Asia

H9N2 avian influenza viruses (AIVs) have spread into multiple Asian countries and became endemic in domestic poultry populations in some of these countries [18, 19, 20]. Its transnational geographic dispersal and exchange of genetic segments with other subtypes in poultry increase their potential for zoonotic threat to public health [18, 21, 22]. Of note, the multi-segmented H9N2 virus provided its internal gene materials to facilitate the genesis of the novel H7N9 AIVs that caused multiple outbreaks and high mortality in humans since 2013 in China [21]. Hereafter the underlying mechanism behind evolution and spread of the “donator” H9N2 virus raises wide concerns [23].

Poultry trade network is a potential source of avian influenza virus mobility in Asia and may help to explain the multiple introductions of H9N2 AIVs from the same genetic group into different countries [24, 25]. Additionally, free-living birds played a limited role in dissemination of poultry-adapted AIVs for the specific host adaption [26]. The G1-like H9N2 virus isolated in middle eastern countries shares a common ancestor with the virus from China [27], and the genetically related H9N2 viruses in geographic regions separated by long distances suggest a role for migration by poultry trade [28]. Further, the international poultry trade increased in recent decades to meet human demand on the cheap protein from poultry. The asymptomatic poultry carrying this low pathogenetic virus could be neglected during transportation process. Poultry transportation can bring together various host species from different regions in a high-density setting and provides an ideal environment for interspecies virus transmission and theretofore the reassortment of different viral segments [29].

Another potential source of AIV mobility is wild bird movements [30, 31, 32]. In 2005, the outbreak of highly pathogenic AIV H5N1 in wild birds in Qinghai Lake, China and its subsequent rapid dissemination from Asia to Europe and Africa led to a great concern about the role of migratory birds played in virus dispersal [33, 34]. Wild aquatic birds can act as natural reservoirs of influenza A viruses, since infection is often asymptomatic or mild, allowing them to migrate while carrying the virus [35]. The virus from wild bird can spread into the free-ranging poultry by frequent contacts in their sharing areas [36]. Viral transmission between domestic and wild birds commonly occurred within a region while mobile and migratory wild birds could transmit viruses between regions [24]. Although poultry trade may play a major role in H9N2 virus spread, the contribution of bird migration to the long-distance virus dispersal between poultry populations should therefore not be neglected. Nevertheless, we here focus on assessing the role of poultry trade and production in the prevalence and spread of H9N2 AIVs.

By elucidating the evolutionary dynamics and the underlying factors that drive virus spread, we aim to better understand the ecology and spread mechanism of the viruses in Asia. In this study, we also described the spatial distribution and temporal dynamic of H9N2 virus isolates. The migration dynamics and their underlying mechanism of H9N2 AIVs between 12 Asian countries and regions were inferred by using two phylogeographic methods DTA and MASCOT in a Bayesian Markov chain Monte Carlo (MCMC) inference framework. Under MASCOT, we also jointly inferred predictors of the effective population size of the virus in each location, which is not possible in DTA. To do so, we used a GLM approach to parameterize migration rates and effective population sizes of H9N2 viruses by potential predictors, including time-dependent predictors (annual live poultry trade, annual national poultry production, yearly mean temperature, yearly total rainfall, annual seasonality of temperature and rainfall, and yearly virus sample size) and time-independent predictors (e.g. geographic distance). The underlying mechanisms of virus migration and genetic diversity were evaluated and the sensitivity of our results was investigated by comparing models that included different predictors, different sub-sampling strategies of genetic data and alternative phylogeographic modelling assumptions.

## Materials and methods

### Sequence data

We obtained all full-length haemagglutinin (HA) segment nucleotide sequences of avian-origin H9N2 from Asian countries and regions that were available in the GenBank Influenza Virus Database (http://www.ncbi.nlm.nih.gov/genomes/FLU/FLU.html) hosted by the National Center for Biotechnology Information (NCBI). Identical sequences caused by duplicated submissions in the database (i.e. same sequence and same isolate name), were reduced to a single sequence to avoid bias in rate estimates. Sequences without explicit isolation date or country information were excluded. These HA sequences from 1976 to 2014 were geocoded and pooled into groups according to their geographic location, host type (chicken, duck, quail, turkey, wild bird, and others), and isolation date (S5 Table).

The virus sequences were aligned in MAFFT v7 software with default parameters [37]. We evaluated the temporal signal of the remaining heterochronous sequences with TempEst [38] and removed sequences that we identified as outliers. To get a more even distribution of samples through time and between different locations, we randomly sub-sampled the H9N2 sequences to keep at most 10 isolates per country/region per year. In order to avoid over-parameterization we discarded locations with less than 10 isolates in total. Finally, we added commonly used representative HA gene sequences to help the phylogenetic clade classification (A/quail/Hong Kong/G1/97 represents the G1 lineage; A/chicken/Hong Kong/G9/97 or A/duck/Hong Kong/Y280/97 or A/chicken/Beijing/1/94 represents the G9 lineage; and A/chicken/Korea/38349-p96323/96 or A/duck/Hong Kong/Y439/97 represents the Korea lineage) [39]. The final data set contained 526 HA sequences from 12 Asian countries/regions. We defined distinct locations on the country/region level as Bangladesh, mainland China, Hong Kong, South Korea, Japan, Vietnam, India, Pakistan, Iran, Israel, Saudi Arabia, and the United Arab Emirates. A dataset of this size is currently an upper limit of what can be analysed by using structured coalescent methods. In order to test the impact of sub-sampling strategy on our inferences, we additionally sub-sampled this dataset by randomly sampling at most 5 isolates per country/region per year, which resulted in a dataset of 385 HA sequences. More detail information on the viral sequences is provided in S5 Table.

### Empirical predictors

To inform the spread and genetic diversity of H9N2 viruses across the 12 locations, we chose several potential predictors. These are similar to ones previously used to describe the spread of H3N2 [9] or Ebola [13, 8]. We used annual live poultry trade, annual poultry production, gross domestic product values (GDP), geographic distance, a predictor describing if countries share a continental border, temperature, temperature seasonality, rainfall, rainfall seasonality, virus sample size and the latitude of centroid point of each country. All the country-level predictors were available from 1986 to 2013.

We downloaded live poultry trade and poultry production data (including data related to chicken, duck, and turkey) from FAOSTAT (http://faostat3.fao.org). Typically, poultry movements are driven by variation in the supply and demand for poultry, which are in turn commonly affected by economic, ecological and climatic conditions [40]. These, as well as practical considerations led us to chose the set of potential predictors used in this study.

GDP statistics were collected to describe the economic level of each country. Annual mean temperature and annual total rainfall were gathered to describe the climatic condition in each country through time. Further, the annual variation of temperature and rainfall were described by temperature seasonality and rainfall seasonality respectively. Temperature seasonality is the standard deviation of the monthly mean temperatures in each year. Rainfall seasonality (*R*_*S*_) for year *t* is the ratio of the standard deviation of the monthly total precipitation (*s*_*p*_) over one plus the mean monthly precipitation (*p*_*m*_): *R*_*S*_(*t*) = *s*_*p*_(*t*)/(1 + *p*_*m*_(*t*)). GDP, temperature, and rainfall data were collected from the World Bank database (http://data.worldbank.org/).

The H9N2 isolates sampled from the same region commonly tended to gather in a phylogenetic group inferred by the Bayesian or maximum likelihood inference [23, 28, 41]. The geographic distance between each pair of locations was considered a potential factor of virus spread and calculated by the great circle distance based on the central latitude and longitude of each location. We also used a predictor with 1 or 0 to describe if two locations share their border on the continent or not, respectively. To test the impact of sampling effects, the number of H9N2 sequences in both origin and destination location was considered as two separate predictors. Finally the geographic central latitudes of locations were considered as a predictor to investigate latitude as a potential driver of H9N2 spread.

To avoid excessive co-linearity among explanatory predictors, we removed the GDP, temperature seasonality and the latitude variables, since the Pearson correlation coefficients between temperature variable and each of them exceeded 0.7. To eliminate the effect of the magnitude of different predictors, all predictors (except binary predictors) were transformed into log space and standardized so that their means are equal to 0 and standard deviation equals to 1. This is standard practice when using generalize linear models to inform viral migration rates and effective population size (e.g. [9]). More detail information on predictors is supplied in S2 Table.

### The DTA model

The DTA model treats movement of viral genes across discrete geographic locations as a continuous time Markov process in which the state space consists of the sampled locations [1, 42]. This model treats the spread of viruses as statistically equivalent to the evolution of molecular substitutions at a site. The posterior probability distribution of parameters given data in the DTA model is shown in Eq 1 [1]. Here, the aligned sequences *S* and the sampling locations *L* are treated as observations, whereas the isolation dates of the sequence *t* are treated as boundary conditions. The phylogenetic tree *T*, the nucleotide substitution rate matrix *μ*, the forwards-in-time migration rate matrix *f* and the effective population size *θ* of the whole meta-population are random variables estimated in this model. The first term on the right is the likelihood of the sequences. This likelihood is calculated by integrating over all possible substitution histories using the pruning algorithm [2]. The second term is the likelihood of the sampling locations given the time-stamped genealogy and the instantaneous migration rate matrix. It is calculated by the same pruning algorithm, but using a migration rate matrix rather than a substitution matrix. The third term describes the probability density of the genealogy across the entire meta-population, approximated by a standard neutral coalescent prior for a well-mixed and unstructured population. The fourth term represents the prior distribution of three independent random variables. It should be noted that to the extent that *θ* can be interpreted in this model, it represents the effective population size for the entire meta-population across all locations in the dataset.

$$\begin{array}{c} P(T,\mu ,f,\theta |S,L)\propto \mathrm{Pr}(S|T,\mu )\mathrm{Pr}(L|T,f)P(T|\theta )P(\mu ,f,\theta ) \end{array}$$ (1)

### The structured coalescent model

The structured coalescent jointly models how lineages coalesce within locations and migration between them. The posterior distribution of the parameters given the data in a structured coalescent phylogeographic inference is described in Eq 2. Here, the meaning of parameters is the same as in the DTA model. However, migration is parameterized as a backwards-in-time migration rate matrix (*m*) and the effective population size $\vec{\theta }$ is modelled separately for each sub-population. The first term is the likelihood of sequences given genealogy and substitution model, which is computed using the pruning algorithm [2]. The second term is the probability density of the genealogy and migration history of lineages under the structured coalescent assumption given the migration rate matrix and effective population sizes. The third term represents the prior distribution of the model parameters.

$$\begin{array}{c} P\left(T,M,\mu ,m,\vec{\theta }|S,L\right)\propto P\left(S|T,\mu \right)P\left(T,M|L,m,\vec{\theta }\right)P\left(\mu ,m,\vec{\theta }\right) \end{array}$$ (2)

The structured coalescent likelihood can be computed analytically only when conditioned on a migration history (*M*). Thus standard approaches have required augmenting the tree with a random-dimensional migration history which has restricted the application of this model to datasets with a small number of demes/locations [5]. However, when the details of the migration history are not of particular interest, the MASCOT model [7] can be used to approximate the integrated likelihood (i.e. formally integrating over every possible migration history for each tree in the MCMC chain). This approximation is closely related to the exact structured coalescent, but still allows us to analyse a dataset with many different sub-populations. Since we seek to investigate the spread of H9N2 between 12 different countries/regions, we used MASCOT in our analyses.

### Migration rate GLMs

DTA and MASCOT have both been extended such that constant migration rates and time-series migration rates can be described using GLMs [7, 9, 17]. This allows us to infer the contribution of explanatory factors to migration rates between different sub-populations, and through time. We examined different sub-sampling scheme on our genetic data, and the effect of including and excluding isolate sample size as a predictor separately in our GLM models. We used a prior probability distribution on the number of active predictors such that 50% of the probability mass is no predictors being included in the GLM. Four variants were considered in GLM under DTA model to investigate the contribution of drivers to the constant migration rates among these unstructured sub-populations; both time-dependent and time-independent predictors were considered to model viral migration rates between different sub-populations in six GLMs using MASCOT (S1 Table).

Migration rates between locations are defined as log-linear combinations of coefficients, indicators, and predictors. Eqs 3 and 4 describe the time-independent and time-dependent parameterizations of the migration rates respectively. Here, *m*_*ij*_ represents the migration rates between location *i* and *j*; $p_{m\{ij\}}^{k}$ represents the *k*-th predictor between location *i* and *j*; $I_{m}^{k}$ represents the indicator and $\beta_{m}^{k}$ represents the coefficient of the *k*th predictor. The indicator and coefficient describe if and to what degree each predictor contributes to explain the migration rates. Indicators are estimated by a Bayesian stochastic search variable selection (BSSVS) algorithm to describe the posterior inclusion probability for each predictor and use the priors on the number of active predictors to reduce over-fitting [43]. Further, *m*_*ij*_(*t*), $p_{m\{ij\}}^{k}\left(t\right)$, $\beta_{m}^{k}\left(t\right)$ and $I_{m}^{k}\left(t\right)$ represent the time-dependent version of corresponding parameters in this model. Because more than 10 predictors were chosen to model the migration rates, we removed the error terms in the GLM model to avoid having to infer too many parameters.

$$\begin{array}{c} \log m_{ij}=\sum_{k=1}^{n}I_{m}^{k}\beta_{m}^{k}\log p_{m\{ij\}}^{k} \end{array}$$ (3)

$$\begin{array}{c} \log m_{ij}\left(t\right)=\sum_{k=1}^{n}I_{m}^{k}\beta_{m}^{k}\log p_{m\{ij\}}^{k}\left(t\right) \end{array}$$ (4)

In contrast to DTA, the structured coalescent models a process from the present backwards in time to the past. Therefore, all parameters of the model are backwards in time parameters, including the migration rates. To compute backwards in time migration rate $m_{ji}^{b}\left(t\right)$ from forwards in time migration rates, we scale the forwards in time rates using the effective population sizes of the source and sink populations:

$$\begin{array}{c} m_{ji}^{b}\left(t\right)\approx \frac{Ne_{i}\left(t\right)}{Ne_{j}\left(t\right)}m_{ij}^{f}\left(t\right) \end{array}$$ (5)

### Effective population size GLMs

Effective population sizes within different sub-populations were modelled by both time-independent (Eq 6) and time-dependent (Eq 7) GLM models in MASCOT. Here, *Ne*_*i*_ represents the viral effective population size of location *i*; $p_{Ne\{i\}}^{k}$ represents the *k*th (time-independent) predictor at location *i*; $\beta_{Ne}^{k}$ and $I_{Ne}^{k}$ represent the coefficient and inclusion probability of the *k*th predictor respectively. *α*_*i*_ represents the extra part of viral effective population size that could not be explained by the predictors in region *i*. Further, *Ne*_*i*_(*t*), $p_{Ne\{i\}}^{k}\left(t\right)$, $\beta_{Ne}^{k}\left(t\right)$ and $I_{Ne}^{k}\left(t\right)$ represent the time-dependent version of corresponding parameters from 1986 to 2013 in the model.

Eq 8 describes a specific instance of the model in Eq 7. The first part of Eq 8 describes the relationship between viral effective population size and poultry production in each region jointly. Here, the number of predictors *n* is equal to the number of locations in the analysis. $P_{Ne\{i\}}^{i}\left(t\right)$ represents the poultry production in region *i* from 1986 to 2013. In order to model the time before 1986 as a structured coalescent process with constant rates, we introduce predictors that are 1 for any of the locations before 1986 and 0 otherwise. One predictor therefore only predicts the *Ne* after 1986 and for only one location. This we do in order to avoid events that happened more than 28 years ago for which we do not have predictor information to bias our inference.

$$\begin{array}{c} \log Ne_{i}=\sum_{k=1}^{n}\left(I_{Ne}^{k}\beta_{Ne}^{k}\log p_{Ne\{i\}}^{k}+\alpha_{i}\right) \end{array}$$ (6)

$$\begin{array}{c} \log Ne_{i}\left(t\right)=\sum_{k=1}^{n}\left(I_{Ne}^{k}\beta_{Ne}^{k}\log p_{Ne\{i\}}^{k}\left(t\right)+\alpha_{i}\right) \end{array}$$ (7)

$$\begin{array}{c} \log Ne_{i}\left(t\right)=\{\begin{array}{cc} \sum_{i=1}^{n}I_{Ne}^{i}\beta_{Ne}^{i}\log P_{Ne\{i\}}^{i}\left(t\right)+\alpha_{i} & t=1,\ldots ,27,\\ \sum_{i=1}^{n}\alpha_{Ne\{i\}} & t=28. \end{array} \end{array}$$ (8)

### Parameter inference

Bayesian analyses of H9N2 AIVs using the DTA and DTA GLM models were conducted using BEAST v1.10.0 [11]. The MASCOT GLM analyses on the same data were conducted using BEAST v2.5.2 [44] and Coupled MCMC [45]. An HKY nucleotide substitution model with gamma site heterogeneity using 4 rate categories and a strict molecular clock model were employed to model sequence evolution in all analyses. The discrete phylogeographic analysis using DTA with asymmetric trait substitution model and Bayesian skyline tree prior was performed in 5 parallel runs. The convergence and mixing of MCMC chains in these runs were diagnosed by the RWTY package in R v3.4.3 [46]. DTA and MASCOT GLM models were used to estimate the contribution of potential predictors to the migration rates between each pair of locations. Further, the MASCOT analyses included population dynamic GLMs to identify the underlying factors driving virus population diversity in each sub-population. We performed at least 5 runs with 10-100 million iterations (based on the convergence time of different analyses) of different analyses to estimate the phylogenetic tree with location information and GLM parameters. We used Tracer v1.7 [47] to remove an appropriate burn-in (10%-20% of samples in most cases) to achieve an adequate effective sample size (ESS, at least 100). The time scaled phylogenetic tree with the maximum probable location in each lineage was annotated and visualized in FigTree v1.4.3 (http://tree.bio.ed.ac.uk/software/figtree/) and the ggtree package in R v3.4.3 [48].

## Results

### H9N2 was endemic in domestic poultry in Asian countries

**Fig 1:**
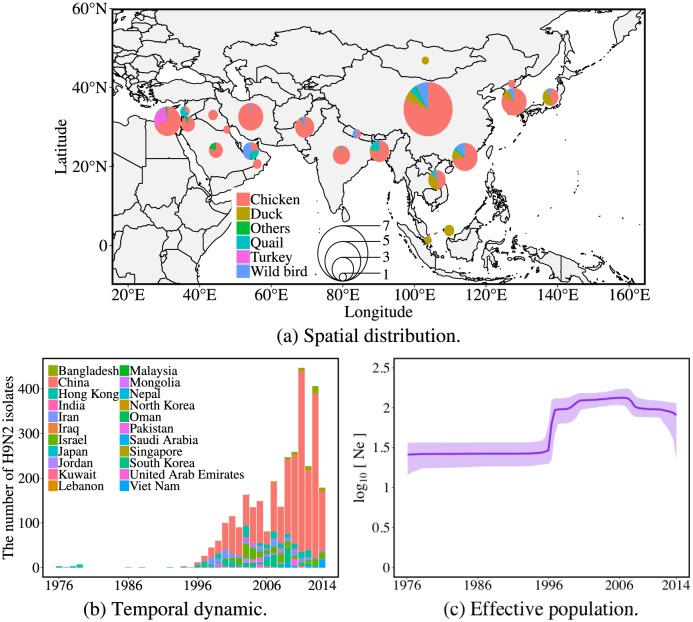
Spatio-temporal distribution of avian-origin H9N2 influenza viruses in Asia between 1976 and 2014. (a) Spatial distribution where the size of circle represents the number of virus isolates obtained in the affected countries/locations. The value of circle indicates double square roots of virus isolate quantity in each location. Colors in the circle represent different fraction of virus hosts. Most of the isolates originate from chickens and from China. The source of map is http://www.naturalearthdata.com. (b) The number of H9N2 HA isolates that are deposited in NCBI through time. The bars represent the annual number of H9N2 isolates sampled in each region. (c) Inferred effective population sizes of H9N2 in Asia by using Bayesian skyline plot. The effective population size of H9N2 viruses increased in 1996 when more isolates began to be sampled in more countries representing multiple outbreaks.

H9N2 viruses spread to at least 22 countries in Asia between 1976 and 2014 (Fig 1a). These 22 countries are mostly located in tropic and temperate zones with lower latitudes, where the climatic conditions are suitable for poultry rearing. H9N2 viruses have been isolated from a wide variety of different hosts, including the major poultry species: chickens and ducks. Compared to the number of isolates from wild birds, H9N2 viruses were predominantly isolated from domestic poultry populations in Asia, especially from chicken. Since 1996, H9N2 viruses have been isolated in more Asian countries and then persisted in birds in some of these countries for several years (Fig 1b). The number of isolates shows an increasing trend and most were sampled from mainland China since the late 1990s, which is likely in part driven by a larger surveillance effort. The estimated effective population size of the virus however also substantially increased since 1996 (Fig 1c).

### Viral phylogeny and dynamics of spread

**Fig 2:**
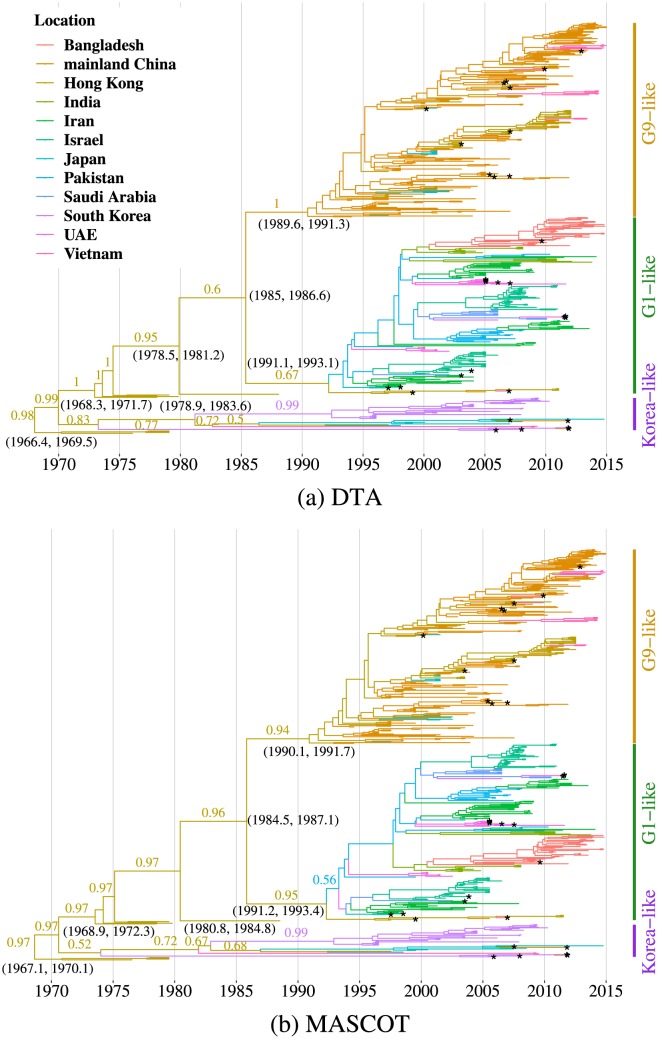
Time scaled phylogenetic trees of H9N2 influenza viruses in Asia. (a) estimated using DTA model and (b) using MASCOT model. The colour of tree branches indicates location (see legend) with the maximum probability. A colour change on a branch indicates a virus migration event. Numbers on branches represent posterior probability of displayed location. Numbers in parentheses represent 95% highest posterior density interval of divergence time of the nearest node. A black asterisk represents a virus sequence isolated from wild bird. UAE is short for the United Arab Emirates. Both methods place the origin of H9N2 in Hong Kong, from where it spread to East Asia. This is likely driven by a lack of samples from other locations in the 1970s and 80s. DTA and MASCOT differ in the details on how it spread to West and South Asia. Bars on the right indicate three established lineages based on the phylogenetic relationship between the virus and the representative strains in Asia. The phylogenetic cluster of isolates from domestic poultry in nearby regions indicates their roles in virus spread among neighbouring locations; whereas the dispersal distribution of isolates from wild birds on the phylogeny questions their roles in virus spread across countries and genetic groups.

The evolutionary relationships and the migration history of lineages reconstructed using the two phylogeographic methods are shown in Fig 2. As mentioned previously, the genealogies can be grouped into three lineages: G9-like, G1-like and Korea-like lineage [39]. These three lineages were established in the 1990s and continue to be isolated to date. Most isolates used in this study were found to be G9- or G1-like. Isolates from countries close by to each other were often genetically related (Fig 2). G9-like H9N2 viruses were mainly isolated in mainland China, Hong Kong, Japan, and Vietnam. G1-like viruses were mainly isolated in the Middle East and the Indian subcontinent. Korea-like viruses were predominantly isolated in South Korea, Japan, and Hong Kong.

Hong Kong was inferred as the most likely source of H9N2 viruses circulating in Asia by both DTA (98% posterior probability) and MASCOT (97% posterior probability) (Fig 2). This is however likely driven by a lack of samples from other regions, for example mainland China, in this era. DTA inferred H9N2 to have spread from Hong Kong to East Asia in the 1980s. After, one part of the viral population continued to spread in countries in the East and Southeast Asia; Another part of the population was transmitted to several countries in the West and South Asia. MASCOT inferred that H9N2 viruses were directly transmitted into countries in the West and South Asia from Hong Kong, and via multiple introductions from Hong Kong to East Asia.

**Fig 3:**
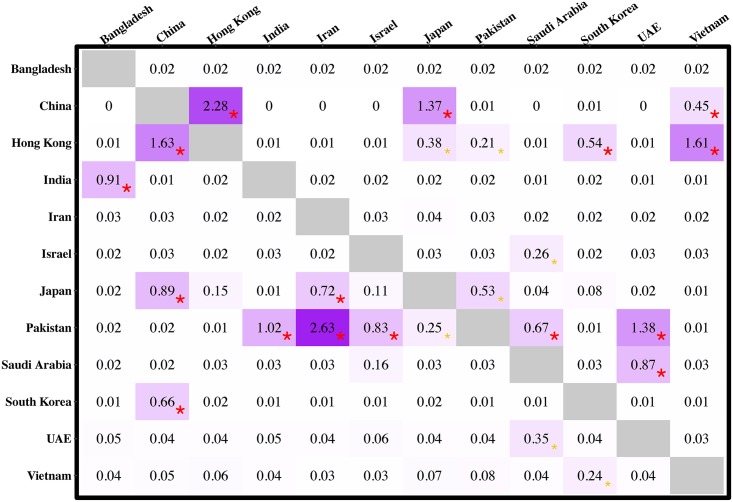
Asymmetric migration rate matrix of H9N2 influenza viruses between each pair of locations in Asia. The migration rate matrix was estimated using DTA, and it describes the virus migration rates between each pair of locations. Unit is the number of migration events per lineage per year. Bayes factors on migration rate over 3 and 20 are labeled by a yellow and a red asterisk at the bottom right of the cell respectively. UAE is short for the United Arab Emirates. The largest and most well-supported rates are between neighbouring locations, suggesting the underlying factors related to geographic proximity can contribute to virus spread.

Viral migration rates among Asian countries and regions were inferred by using DTA with asymmetric migration rates (Fig 3). The highest migration rates with the strongest support were inferred from Pakistan to Iran, between Hong Kong and mainland China, from Hong Kong to Vietnam, from Pakistan to the United Arab Emirates, from mainland China to Japan, from Pakistan to India, and from India to Bangladesh. All these pairs are locations within close geographic proximity. The migration rates among these pairs are 0.91 or higher, which means ∼ 1 or more migration event occurred between these locations per lineage per year. Additionally, the phylodynamic reconstructions on root state, location transition dynamics in evolutionary history, and migration rates of H9N2 are broadly consistent when we employed the sub-sampling pattern including 385 or 526 HA sequences(S1 and S2 Figs).

### The mechanisms of virus spatial spread

**Fig 4:**
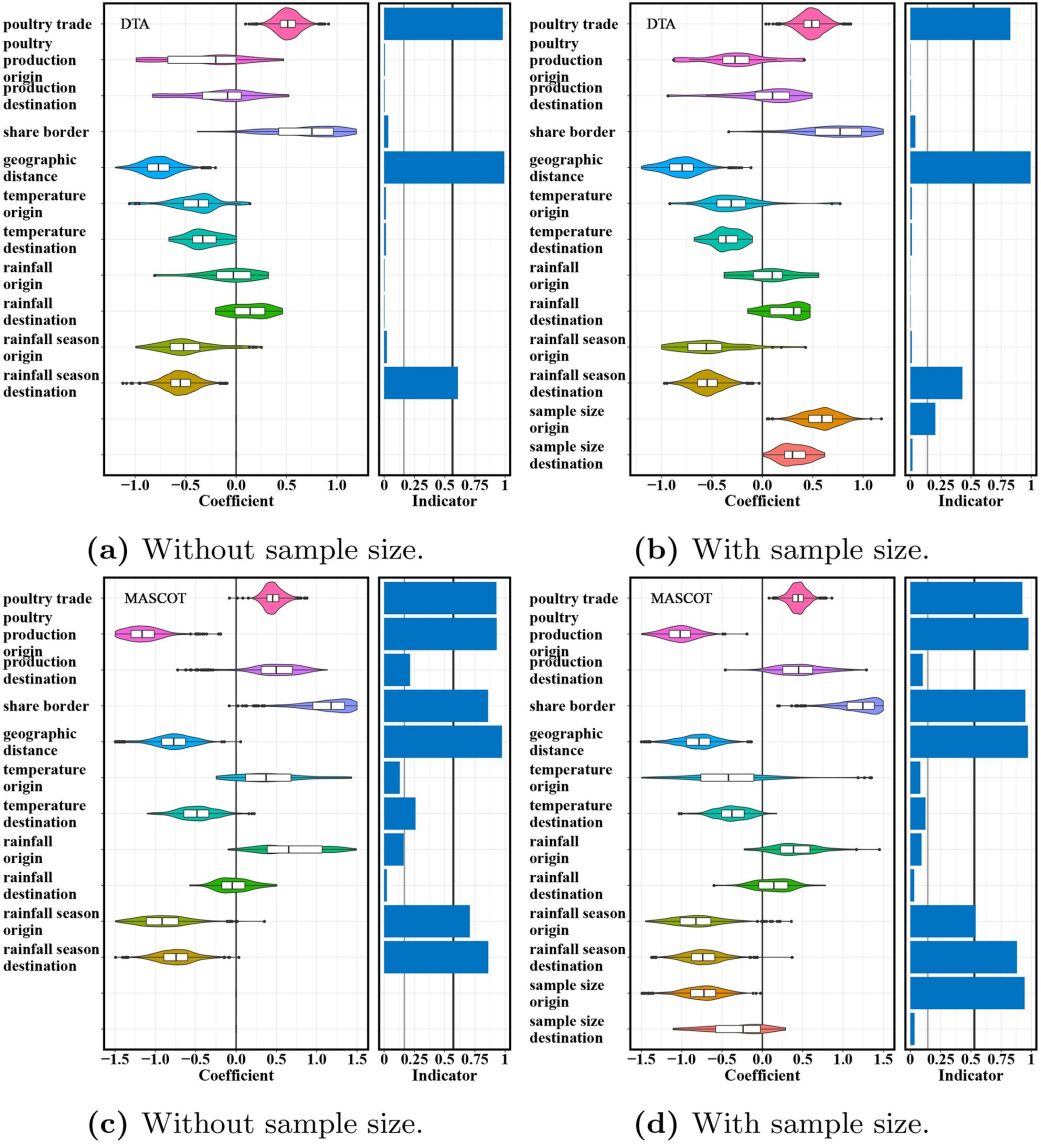
Predictors of migration rates of H9N2 influenza viruses between 12 countries/locations in Asia. The estimated coefficients and inclusion probabilities for potential predictors of migration rates in the DTA model: (a) without and (b) with isolate sample sizes included as potential predictors; in the time-dependent MASCOT GLMs: (c) without and (d) with isolate sample sizes considered as a predictor. The 50% prior mass was specified on no predictors being included. Coefficients represent the contribution of each predictor to the migration rates of H9N2 AIVs when the corresponding predictor was included in the model. Inclusion probabilities are calculated by proportion of the posterior samples in which each predictor was included in the model. Bayes factor support values of 3 and 20 are represented by a thin and thick vertical line respectively in the inclusion probabilities plot. Geographic distance, poultry trade and rainfall seasonality in destination are the most strongly supported factors to virus spread in Asia under cross-validation in these models. Sample size at origin has an effect, but it doesn’t change the support of other predictors.

We next investigated the factors driving the spread of H9N2 viruses across several locations in Asia by using a GLM to model the relationship between potential predictors and viral migration rates (Fig 4, S3 and S4 Figs). A total of 10 different GLMs were used (S1, S3 and S4 Tables). We used both DTA and MASCOT and included time-dependent and time-independent predictors; 385 or 525 HA sequences. Further, we ran analyses with and without considering the number of isolates as a distinct predictor. Bayes factors (BFs) on inclusion probability of each predictor were calculated to explain how much the data informed the inclusion of a predictor [49]. BF is calculated as a ratio of the posterior odds for a predictor inclusion to the corresponding prior odds. A BF over 3 is typically considered suggestive and a BF over 20 is typically used as strongly supporting a predictor to be included into the GLM model [50]. In addition, we also evaluate the robustness and support of a predictor can inform virus migration rates by counting times each predictor was selected as a supportive one in all migration rate GLMs investigated. If a predictor was always selected as supportive regardless of the heterogeneous sampling intensity and model assumption, it can robustly inform virus spread.

Geographic distance was identified as a strongly supported driver in all migration rate GLMs investigated and it consistently made a negative contribution to the virus spread (S4 Table), meaning that migration is inferred to be stronger between closer countries. We inferred poultry trade to strongly predict viral migration in all GLMs except the time-independent ones under MASCOT. Predictors related to rainfall seasonality in destination location and boarder sharing were also inferred in more than half of all GLMs investigated to be strongly supported. Additionally, we inferred migration to be weaker into locations with strong seasonality in rainfall. Further, our time-dependent GLM of MASCOT was more sensitive as identified by predictor poultry production in origin and rainfall seasonality. These results were consistent and robust when we included viral sample size as a distinct predictor or when we used fewer samples (S4 Table).

### Predictors of viral effective population sizes through time

**Fig 5:**
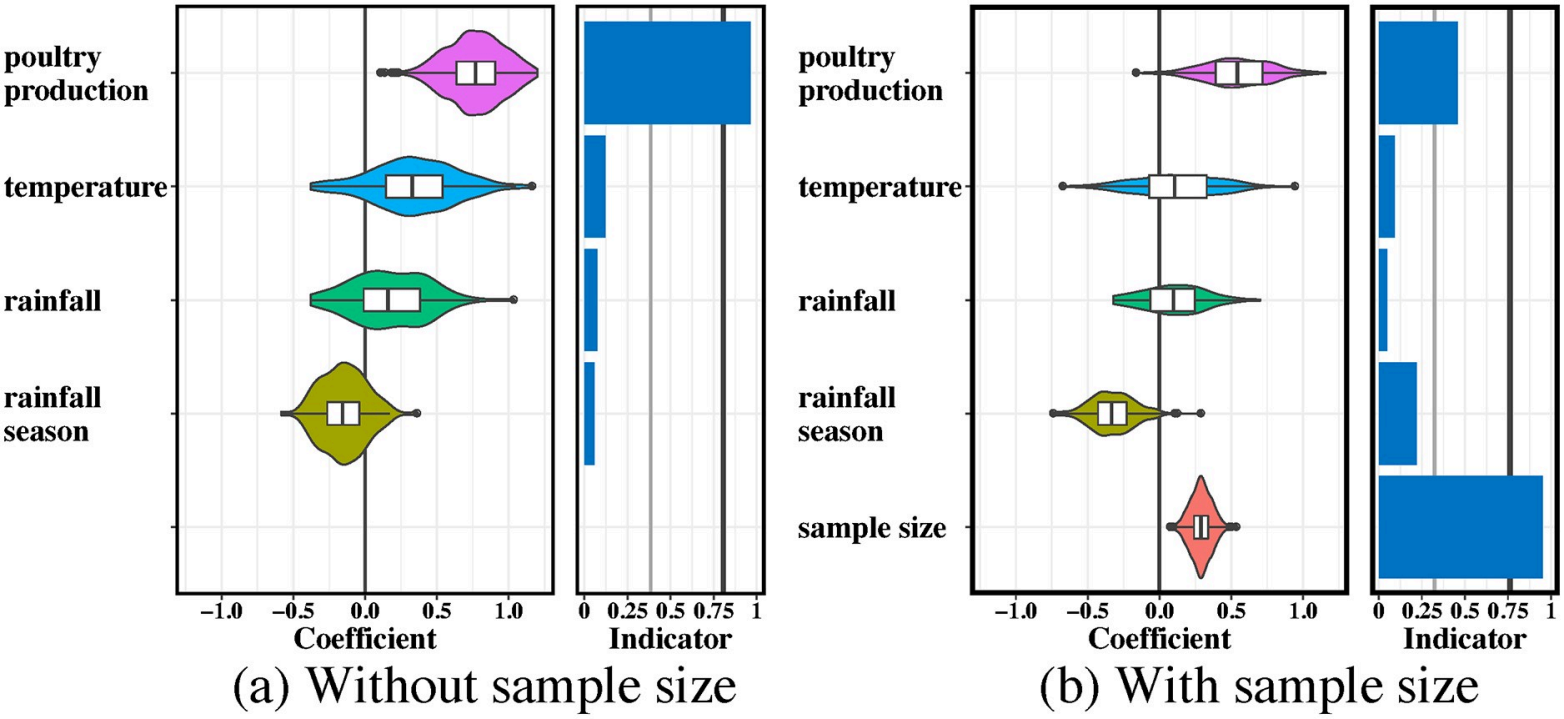
Predictors of time-dependent population dynamics of H9N2 influenza viruses within 12 countries/locations in Asia. Coefficients and indicators as in Fig 4 when estimating the effective population size of H9N2 AIVs (a) without and (b) with considering the effect of virus sample size included as a distinct predictor. The 50% prior mass was specified on no predictors being included. Bayes factor support values of 3 and 20 are represented by a thin and thick vertical line respectively in the indicator plot. Poultry production positively contributes to virus population size. When the number of samples through time in each location is also used as a predictor, the effect of poultry production is much less pronounced for the virus sampling may have been approximately proportional to effective population sizes.

In MASCOT, we also jointly inferred predictors of effective population sizes of H9N2 virus in the different locations and their changes through time. The effective population sizes in the different locations were modelled by multiple time-independent and time-dependent predictors in GLMs (Fig 5, S5 and S6 Figs). Poultry production was selected as a supportive predictor for the effective population size of H9N2 virus in GLM model with time-dependent predictors (Fig 5). This implies that the higher the poultry production in a country is, the larger the genetic diversity of H9N2 virus in that country. Furthermore, virus sample size was also considered as a positive and supportive predictor, and the inclusion of virus sample size into the GLM model lowered the contribution of poultry production to virus population diversity. This suggests that the number of viral samples through time was roughly proportional to the effective population sizes. No potential predictors of effective population sizes were supported when we assumed that the effective population sizes are constant through time (S6 Fig). Additionally, we applied a GLM that used time-dependent poultry production data in 12 Asian countries or regions to model the virus population dynamics in each sub-population jointly (S7 Fig). Poultry production in mainland China was strongly supported as a positive predictor of the local viral effective population size, but not in any other location.

## Discussion

In Asia, H9N2 viruses have spread into multiple countries which did not previously have documented viral isolation in the 1990s and persist in domestic poultry in some of these countries [18, 19, 20]. To reduce the cost and maximize the profit, poultry farms are often built in close proximity to one another and rearing facilities tend to be overcrowded [51]. Viruses can therefore spread easily and outbreaks in poultry pose great economic threats to some of these countries. Further, most of these countries are low- and middle-income countries with poor bio-security. Low sanitary standards and high density of poultry in farms and markets can additionally facilitate the transmission of viruses [52]. Multiple influenza subtypes simultaneously circulate in birds in these countries [28, 54], which increases the probability of reassortment of influenza segments.

In this work, we investigated the evolutionary dynamics and the spread of avian influenza H9N2 in Asia and attempted to uncover factors that potentially predict this spread by using a GLM in two phylogeographic frameworks, DTA and MASCOT [9, 8]. Alongside estimating the factors driving migration rates, we also jointly investigated potential drivers of virus effective population sizes in MASCOT. To do so, we used H9N2 viral HA sequences isolated from avian hosts and 12 locations in Asia between 1976 and 2014. We used different predictor data to inform the viral migration rates between 12 countries/locations. These predictors however ignore other potential drivers of migration, such as wild bird migration, and different sanitation levels among countries. Typically, predictors adopted to explain the virus spread and diversity were scale-dependent. In the future, more exact and more high-resolution predictors could be included to test more detailed hypotheses and model influenza movements in a smaller and confined geographical region [9].

Since rate estimates of DTA are likely to be sensitive to the number of sequences sampled in each location [3], we repeated analyses under two sub-sampling strategies. Additionally, we performed the GLM analyses with and without considering the viral isolate sample size in each country or region as a distinctive predictor to test the impact of heterogeneous sampling intensity. The results were mostly robust and consistent whether this predictor was included or not in DTA using 385 or 526 HA sequences. But the inclusion of isolate sample size in the model did reduce the support of the predictor rainfall seasonality in the destination location, which is negatively related to virus migration rates. Further, sharing a border was included as a suggestive predictor in the DTA GLMs with 385 sequences. These slight exceptions can result from the heterogeneous samples across locations or different information variance provided by two genetic data sets.

We found geographic proximity between locations to be a strong driver of H9N2 migration rates in all GLM models investigated. Additionally, we found that whether two locations are neighbouring each other to be a strong predictor of migration. The contribution of geographic proximity to viral spread was intuitively recorded in the close evolutionary relationship among viruses sampled from nearby countries. Further, a consistent role for poultry trade was inferred in both a GLM with time-independent predictors in DTA and a GLM with time-dependent predictors in MASCOT. This suggests that poultry trade is probably a driver of the spread of avian influenza H9N2 viruses. Infected poultry, especially chicken, without strong clinical symptoms can easily be missed during the process of transportation. H9N2 viruses can therefore spread into native poultry. Increased surveillance of imported poultry and their products could decrease the spread of H9N2 across locations [53]. Illegal poultry trade across borders is another potential factor contributing to the spread of H9N2 [54]. However, even when controlling for poultry trade volumes and other potential predictors, we still found geographic proximity to be a key driver to migration rates. This may point to some factors directly linked to geographical distance to contribute to the viral spread of H9N2 across countries. Contact between domestic and wild birds is inevitable in the intensive and outdoor-reared livestock farms and two-way virus transmission has been documented between them [24, 55]. Wild birds could therefore spread H9N2, as they can easily cross borders and then transmit the viruses. The dispersal distribution of H9N2 isolates from wild birds on the phylogenetic tree supports the possibility of their movement facilitating virus spread across countries and across genetic groups (Fig 2). Future studies will however have to investigate if wild bird migration is really associated with the spread of H9N2 viruses. Further, the region with less monthly variance in rainfall volumes could provide stable feeding and habitat areas for birds and attract the birds carrying viruses. Active surveillance of migratory birds could therefore help to monitor the dispersal of H9N2 virus.

Inferences of migration rate GLM variants we investigated can be divergent such that poultry trade has strong support in all GLM models except ones with time-independent predictors in MASCOT. This divergence shows that including time dependence can be important to identify predictors to inform the heterogeneous spatial diffusion the processes through time. In addition, different inferences of GLMs via DTA and MASCOT can result from diverse migration rate definitions and model assumptions. The DTA models migration rate as the frequency of migration events, while the structured coalescent model describes it by virus genetic diversity spreading among different locations [1, 7].

To improve our understanding of what potentially drives genetic diversity of H9N2, we also used a GLM approach to inform effective population sizes of H9N2 virus in each location and through time by using MASCOT [8]. Time-dependent poultry production was identified as a positive driver to virus divergence within each sub-population. When including the number of samples through time, the support for poultry production as an effective population size predictor decreased. This can be caused by a proportional relationship between the annual number of viral samples and the viral effective population size in a location over time. Mainland China was likely the main driver of poultry production being an effective population size predictor. Approximately 78% of H9N2 samples were isolated from China based on the HA gene sequences recorded in NCBI [56]. The positive correlation between an increasing poultry production and an increasing effective population size of the virus suggests that virus control measures in local poultry may not currently be sufficient.

Surveillance of high pathogenic H5N1 AIVs in ducks has been actively carried out in several Asian countries [57]. Samples from chickens, wild bird, and the environment could also be collected to investigate the prevalence of H9N2 and other subtypes. If there are H9N2 cases in humans, having such samples readily available can help to track possible origins of these cases. Additionally, making surveillance of both high pathogenic and low pathogenic AIVs in poultry and humans routine can potentially help to improve our understanding of how these viruses jump into humans. Overall, the integration of temporal predictors into phylodynamics provides a powerful tool to test how disease spread within and between populations.

## Supporting information

S1 Fig Time scaled phylogenetic trees of H9N2 influenza viruses in Asia estimated by 385 HA sequences. (a) estimated using DTA model and (b) using MASCOT model. The colour of tree branches indicates location (see legend) with the maximum probability. A colour change on a branch indicates a virus migration event. Numbers on branches represent posterior probability of displayed location. A black asterisk represents a virus sequence isolated from a wild bird. UAE is short for the United Arab Emirates. Both models place the source of H9N2 in Hong Kong, from where it spread to East Asia. DTA and MASCOT differ in the details on how it spread to West and South Asia. Bars on the right indicate three established lineages based on the phylogenetic relationship between H9N2 viruses and their representative strains.
(PDF)

S2 Fig Asymmetric migration rate matrix of H9N2 influenza viruses between countries/regions in Asia estimated by 385 sequences. The migration rate matrix was estimated using DTA, and it describes the virus migration rates between each pair of locations. Unit is the number of migration events per lineage per year. Bayes factors on migration rate over 3 and 20 are labeled by a yellow and a red asterisk at the bottom right of the cell respectively. UAE is short for the United Arab Emirates. The largest and most well-supported rates are between neighbouring locations, suggesting the underlying factors related to geographic proximity can contribute to virus spread.
(PDF)

S3 Fig Predictors of migration rates of H9N2 influenza viruses between 12 countries/regions in Asia estimated by 385 sequences. Estimated coefficients and inclusion probabilities for potential predictors of migration rates in DTA GLMs: (a) without and (b) with isolate sample size included as a predictor; in the time-dependent MASCOT GLMs: (c) without and (d) with isolate quantity considered as a predictor. The 50% prior mass was specified on no predictors being included. Coefficients represent the contribution of each predictor to H9N2 migration rates of when the corresponding predictor was included in the model. Inclusion probabilities are calculated by proportion of the posterior samples in which each predictor was included in the model. Bayes factor support values of 3 and 20 are represented by a thin and thick vertical line respectively in the inclusion probabilities plot. Geographic distance and poultry trade are identified as strongly supported factors to virus spread in all four GLMs. Including predictor sample size at origin slightly changes the support of some predictors.
(PDF)

S4 Fig Time-independent predictors of migration rates of H9N2 between 12 locations in Asia inferred by 526 sequences under MASCOT. The 50% prior mass was specified on no predictors being included in the GLM. Parameters and figure elements are the same as in S3 Fig.
(PDF)

S5 Fig Time-dependent predictors of effective population dynamics of H9N2 influenza viruses within 12 countries/regions in Asia estimated by 385 sequences. The GLM (a) without and (b) with including virus sample size as a distinctive predictor. The 50% prior mass was specified on no predictors being included in the GLMs. Parameters and figure elements are the same as in S3 Fig. Poultry production and sample size positively contribute to virus effective population size.
(PDF)

S6 Fig Time-independent predictors of effective population dynamics of H9N2 influenza viruses in 12 countries/regions in Asia estimated by 526 sequences. The GLM (a) without and (b) with including virus isolate size as a distinctive predictor. The 50% prior mass was specified on no predictors being included in the GLMs. Parameters and figure elements here are the same as in S3 Fig. No supportive predictor can inform the virus migration rates in these models.
(PDF)

S7 Fig Time-dependent predictor poultry production of effective population dynamics of H9N2 influenza viruses in 12 countries/regions in Asia estimated by 526 sequences. The 50% prior mass was specified on no predictors being included in the GLM. Parameters and figure elements here are the same as in S3 Fig. National poultry production is a strongly supported driver to virus genetic diversity through time in mainland China.
(PDF)

S1 Table Different scenarios of the 10 migration rate GLMs. (PDF)

S2 Table Predictors considered to inform H9N2 migration rates in GLMs. (PDF)

S3 Table Times each predictor was selected with suggestive support in 10 migration rate GLMs. The predictor with BF over 3 was considered as a suggestive support one. The column names except the first two columns showed the name of different migration rates GLM models in this study. In these columns, value 1 in each cell represents the predictor is a suggestive support one in the corresponding model; 0 represents the predictor is not suggestively supported. The total column represents the total times of each predictor was chosen as suggestive support in the 10 GLMs investigated.
(PDF)

S4 Table Times each predictor was selected with strong support in 10 migration rate GLMs. The predictor with BF over 20 was considered as strong support. Parameters here are the same as in S3 Table.
(PDF)

S5 Table Details about H9N2 HA nucleotide sequences used in our analyses. In the column “Label”, D1 points to sequences exclusively in the data set with 526 HA genes; D2 points to sequences simultaneously in both data sets with 526 and 385 HA genes; G1, G9, and Korea represent the representative sequences; C represents outliers in sequences detected by clock test in TempEst; S represents the removed sequences from phylogeographic inference by down-sampling; L represents the removed sequences from phylogeographic inferences in countries with less than 10 isolates in total; P represents the sequences with partial length. All sequences shown here were used in spatiotemporal analysis of H9N2 virus; Sequences labeled by D1, D2 and the representatives were used in phylogeographic reconstructions.
(PDF)
