# Supplementary material for: Bayesian phylodynamics of avian influenza A virus H9N2 in Asia with time-dependent predictors of migration
Source: PLoS Comput Biol. 2019 Aug 6;15(8):e1007189. doi: 10.1371/journal.pcbi.1007189 (PMC6684064; doi:10.1371/journal.pcbi.1007189)
Supplement: S1 Fig — (a) estimated using DTA model and (b) using MASCOT model. The colour of tree branches indicates location (see legend) with the maximum probability. A colour change on a branch indicates a virus migration event. Numbers on branches represent posterior probability of displayed location. A black asterisk represents a virus sequence isolated from a wild bird. UAE is short for the United Arab Emirates. Both models place the source of H9N2 in Hong Kong, from where it spread to East Asia. DTA and MASCOT differ in the details on how it spread to West and South Asia. Bars on the right indicate three established lineages based on the phylogenetic relationship between H9N2 viruses and their representative strains. (PDF) [file pcbi.1007189.s001.pdf]

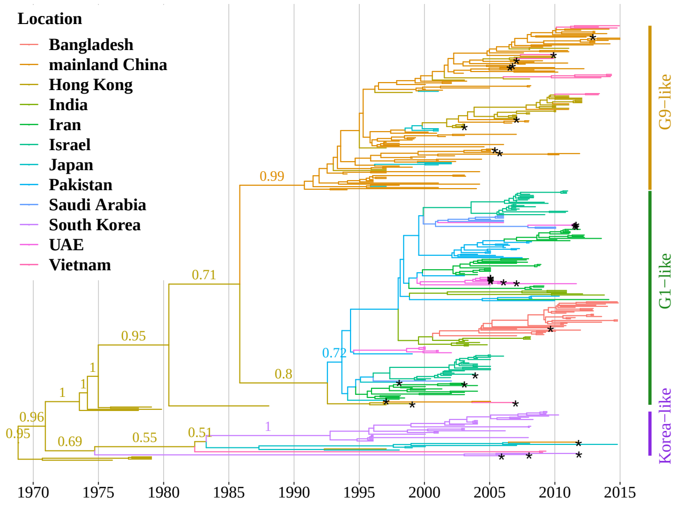

(a) DTA

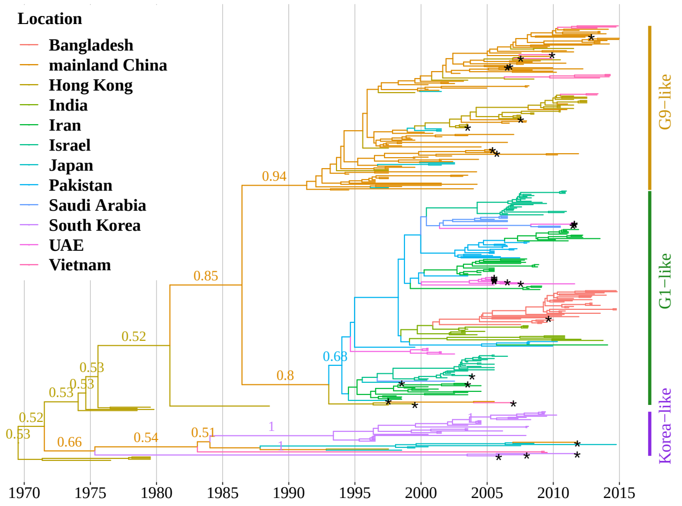

(b) MASCOT

S1 Fig. Time scaled phylogenetic trees of H9N2 influenza viruses in Asia estimated by 385 HA sequences. (a) estimated using DTA model and (b) using MASCOT model. The colour of tree branches indicates location (see legend) with the maximum probability. A colour change on a branch indicates a virus migration event. Numbers on branches represent posterior probability of displayed location. A black asterisk represents a virus sequence isolated from a wild bird. UAE is short for the United Arab Emirates. Both models place the source of H9N2 in Hong Kong, from where it spread to East Asia. DTA and MASCOT differ in the details on how it spread to West and South Asia. Bars on the right indicate three established lineages based on the phylogenetic relationship between H9N2 viruses and their representative strains.
