# Supplementary material for: Bayesian phylodynamics of avian influenza A virus H9N2 in Asia with time-dependent predictors of migration
Source: PLoS Comput Biol. 2019 Aug 6;15(8):e1007189. doi: 10.1371/journal.pcbi.1007189 (PMC6684064; doi:10.1371/journal.pcbi.1007189)
Supplement: S2 Fig — The migration rate matrix was estimated using DTA, and it describes the virus migration rates between each pair of locations. Unit is the number of migration events per lineage per year. Bayes factors on migration rate over 3 and 20 are labeled by a yellow and a red asterisk at the bottom right of the cell respectively. UAE is short for the United Arab Emirates. The largest and most well-supported rates are between neighbouring locations, suggesting the underlying factors related to geographic proximity can contribute to virus spread. (PDF) [file pcbi.1007189.s002.pdf]

	Bangladesh	China	Hong Kong	India	Iran	Israel	Japan	Pakistan	Saudi Arabia	South Korea	UAE	Vietnam
**Bangladesh**		0.02	0.02	0.03	0.02	0.02	0.02	0.02	0.02	0.02	0.02	0.02
**China**	0		2.04 *	0	0	0	1.36 *	0.02	0	0.04	0	0.67 *
**Hong Kong**	0.01	2.22 *		0.01	0.01	0.01	0.2	0.38 *	0.01	0.43 *	0.01	1.43 *
**India**	0.94 *	0.02	0.02		0.01	0.02	0.02	0.02	0.02	0.01	0.02	0.01
**Iran**	0.02	0.02	0.03	0.03		0.03	0.06	0.03	0.02	0.02	0.02	0.02
**Israel**	0.04	0.03	0.03	0.03	0.03		0.05	0.03	0.23 *	0.03	0.03	0.03
**Japan**	0.02	0.58 *	0.77 *	0.01	0.06	0.04		0.21 *	0.02	0.16	0.01	0.03
**Pakistan**	0.02	0.02	0.02	1.05 *	2.08 *	0.87 *	0.61 *		0.74 *	0.02	1.43 *	0.01
**Saudi Arabia**	0.03	0.03	0.02	0.03	0.04	0.15	0.03	0.03		0.02	1 *	0.02
**South Korea**	0.03	0.04	0.03	0.04	0.04	0.03	0.07	0.04	0.03		0.04	0.04
**UAE**	0.04	0.05	0.05	0.05	0.04	0.08	0.05	0.05	0.33 *	0.05		0.05
**Vietnam**	0.03	0.06	0.04	0.04	0.05	0.03	0.13	0.07	0.05	0.31 *	0.04	

S2 Fig. Asymmetric migration rate matrix of H9N2 influenza viruses between countries/regions in Asia estimated by 385 sequences. The migration rate matrix was estimated using DTA, and it describes the virus migration rates between each pair of locations. Unit is the number of migration events per lineage per year. Bayes factors on migration rate over 3 and 20 are labeled by a yellow and a red asterisk at the bottom right of the cell respectively. UAE is short for the United Arab Emirates. The largest and most well-supported rates are between neighbouring locations, suggesting the underlying factors related to geographic proximity can contribute to virus spread.
