# Supplementary material for: Bayesian phylodynamics of avian influenza A virus H9N2 in Asia with time-dependent predictors of migration
Source: PLoS Comput Biol. 2019 Aug 6;15(8):e1007189. doi: 10.1371/journal.pcbi.1007189 (PMC6684064; doi:10.1371/journal.pcbi.1007189)
Supplement: S3 Fig — Estimated coefficients and inclusion probabilities for potential predictors of migration rates in DTA GLMs: (a) without and (b) with isolate sample size included as a predictor; in the time-dependent MASCOT GLMs: (c) without and (d) with isolate quantity considered as a predictor. The 50% prior mass was specified on no predictors being included. Coefficients represent the contribution of each predictor to H9N2 migration rates of when the corresponding predictor was included in the model. Inclusion probabilities are calculated by proportion of the posterior samples in which each predictor was included in the model. Bayes factor support values of 3 and 20 are represented by a thin and thick vertical line respectively in the inclusion probabilities plot. Geographic distance and poultry trade are identified as strongly supported factors to virus spread in all four GLMs. Including predictor sample size at origin slightly changes the support of some predictors. (PDF) [file pcbi.1007189.s003.pdf]

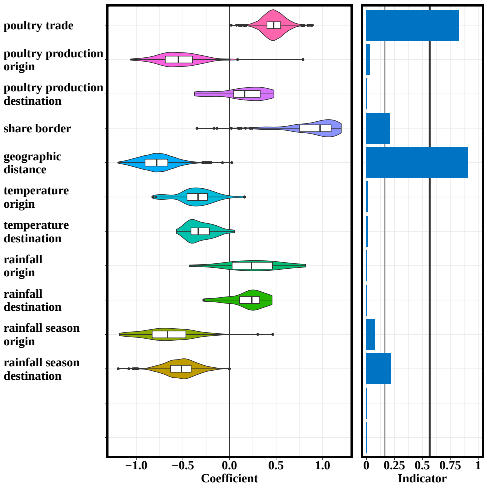

(a) Without sample size.

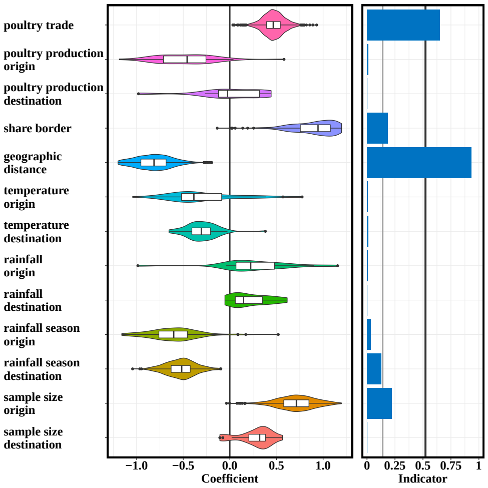

(b) With sample size.

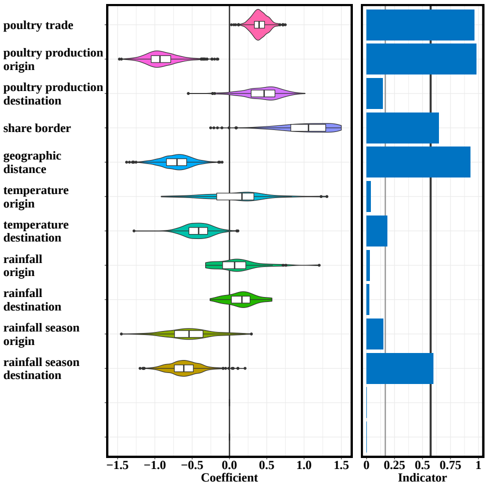

(c) Without sample size.

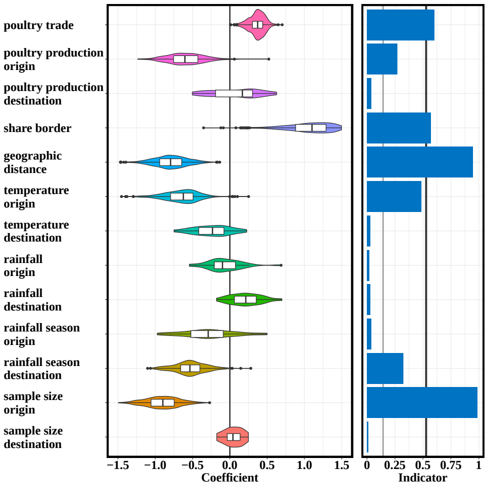

(d) With sample size.

S3 Fig. Predictors of migration rates of H9N2 influenza viruses between 12 countries/regions in Asia estimated by 385 sequences. Estimated coefficients and inclusion probabilities for potential predictors of migration rates in DTA GLMs: (a) without and (b) with isolate sample size included as a predictor; in the time-dependent MASCOT GLMs: (c) without and (d) with isolate quantity considered as a predictor. The 50% prior mass was specified on no predictors being included. Coefficients represent the contribution of each predictor to H9N2 migration rates of when the corresponding predictor was included in the model. Inclusion probabilities are calculated by proportion of the posterior samples in which each predictor was included in the model. Bayes factor support values of 3 and 20 are represented by a thin and thick vertical line respectively in the inclusion probabilities plot. Geographic distance and poultry trade are identified as strongly supported factors to virus spread in all four GLMs. Including predictor sample size at origin slightly changes the support of some predictors.
