# Supplementary material for: Bayesian phylodynamics of avian influenza A virus H9N2 in Asia with time-dependent predictors of migration
Source: PLoS Comput Biol. 2019 Aug 6;15(8):e1007189. doi: 10.1371/journal.pcbi.1007189 (PMC6684064; doi:10.1371/journal.pcbi.1007189)
Supplement: S4 Fig — The 50% prior mass was specified on no predictors being included in the GLM. Parameters and figure elements are the same as in S3 Fig. (PDF) [file pcbi.1007189.s004.pdf]

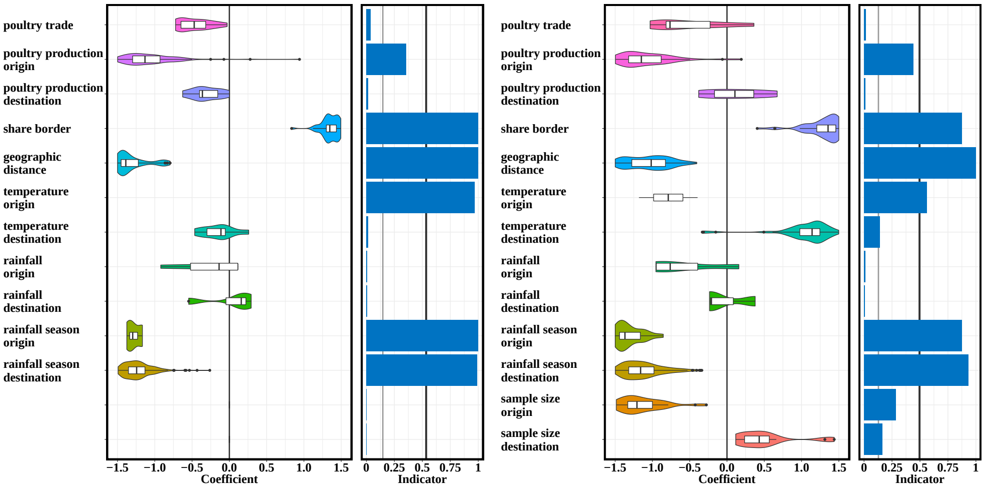

(a) Without sample size. **(b)** With sample size.

S4 Fig. Time-independent predictors of migration rates of H9N2 between 12 locations in Asia inferred by 526 sequences under MASCOT. The 50% prior mass was specified on no predictors being included in the GLM. Parameters and figure elements are the same as in S3 Fig.
