# Supplementary material for: Bayesian phylodynamics of avian influenza A virus H9N2 in Asia with time-dependent predictors of migration
Source: PLoS Comput Biol. 2019 Aug 6;15(8):e1007189. doi: 10.1371/journal.pcbi.1007189 (PMC6684064; doi:10.1371/journal.pcbi.1007189)
Supplement: S5 Fig — The GLM (a) without and (b) with including virus sample size as a distinctive predictor. The 50% prior mass was specified on no predictors being included in the GLMs. Parameters and figure elements are the same as in S3 Fig. Poultry production and sample size positively contribute to virus effective population size. (PDF) [file pcbi.1007189.s005.pdf]

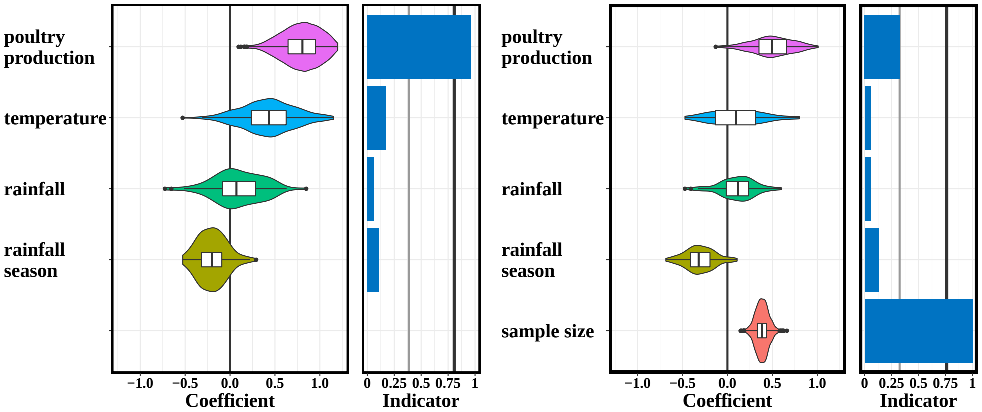

(a) Without sample size.

(b) With sample size.

S5 Fig. Time-dependent predictors of effective population dynamics of H9N2 influenza viruses within 12 countries/regions in Asia estimated by 385 sequences. The GLM (a) without and (b) with including virus sample size as a distinctive predictor. The 50% prior mass was specified on no predictors being included in the GLMs. Parameters and figure elements are the same as in S3 Fig. Poultry production and sample size positively contribute to virus effective population size.
