# Supplementary material for: Bayesian phylodynamics of avian influenza A virus H9N2 in Asia with time-dependent predictors of migration
Source: PLoS Comput Biol. 2019 Aug 6;15(8):e1007189. doi: 10.1371/journal.pcbi.1007189 (PMC6684064; doi:10.1371/journal.pcbi.1007189)
Supplement: S6 Fig — The GLM (a) without and (b) with including virus isolate size as a distinctive predictor. The 50% prior mass was specified on no predictors being included in the GLMs. Parameters and figure elements here are the same as in S3 Fig. No supportive predictor can inform the virus migration rates in these models. (PDF) [file pcbi.1007189.s006.pdf]

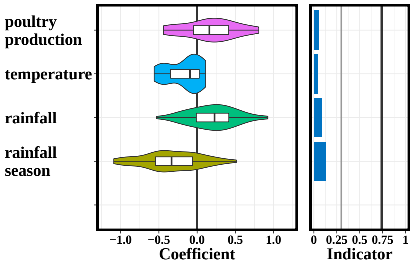

(a) Without sample size.

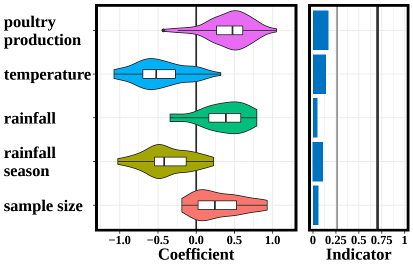

(b) With sample size.

S6 Fig. Time-independent predictors of effective population dynamics of H9N2 influenza viruses in 12 countries/regions in Asia estimated by 526 sequences. The GLM (a) without and (b) with including virus isolate size as a distinctive predictor. The 50% prior mass was specified on no predictors being included in the GLMs. Parameters and figure elements here are the same as in S3 Fig. No supportive predictor can inform the virus migration rates in these models.
