# Supplementary material for: Bayesian phylodynamics of avian influenza A virus H9N2 in Asia with time-dependent predictors of migration
Source: PLoS Comput Biol. 2019 Aug 6;15(8):e1007189. doi: 10.1371/journal.pcbi.1007189 (PMC6684064; doi:10.1371/journal.pcbi.1007189)
Supplement: S7 Fig — The 50% prior mass was specified on no predictors being included in the GLM. Parameters and figure elements here are the same as in S3 Fig. National poultry production is a strongly supported driver to virus genetic diversity through time in mainland China. (PDF) [file pcbi.1007189.s007.pdf]

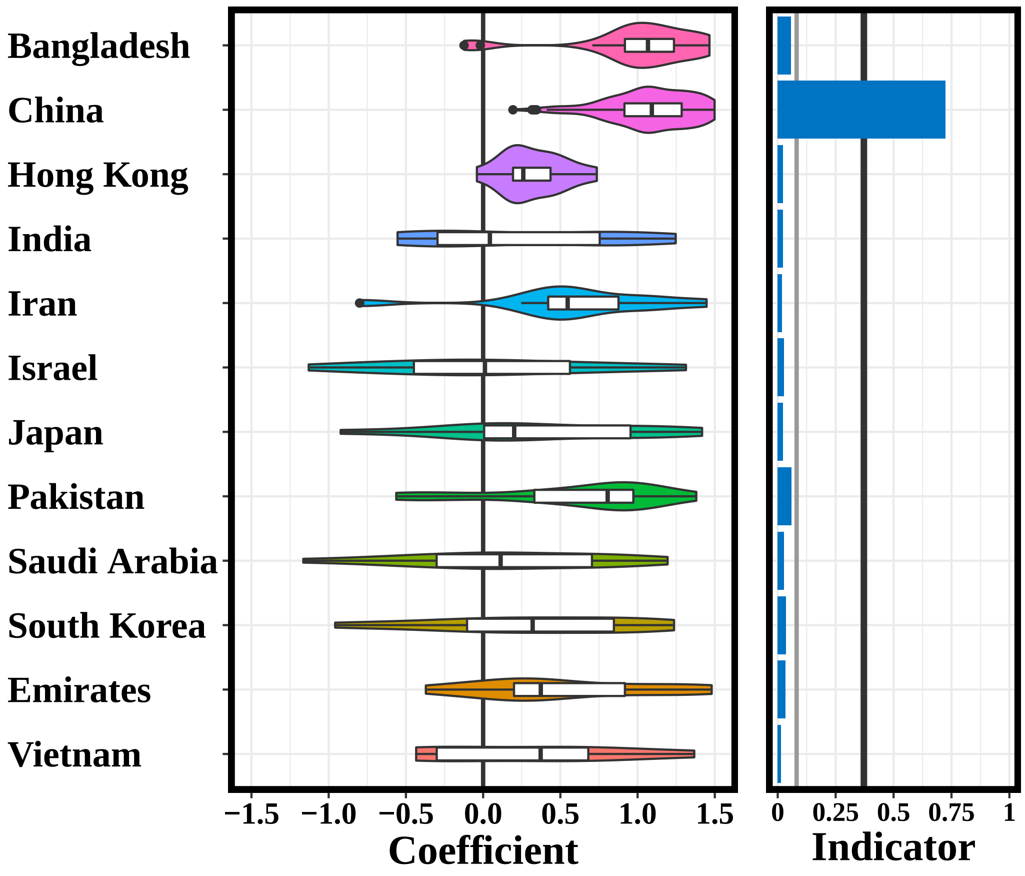

S7 Fig. Time-dependent predictor poultry production of effective population dynamics of H9N2 influenza viruses in 12 countries/regions in Asia estimated by 526 sequences. The 50% prior mass was specified on no predictors being included in the GLM. Parameters and figure elements here are the same as in S3 Fig. National poultry production is a strongly supported driver to virus genetic diversity through time in mainland China.
