# Supplementary material for: Bayesian phylodynamics of avian influenza A virus H9N2 in Asia with time-dependent predictors of migration
Source: PLoS Comput Biol. 2019 Aug 6;15(8):e1007189. doi: 10.1371/journal.pcbi.1007189 (PMC6684064; doi:10.1371/journal.pcbi.1007189)
Supplement: S1 Table — (PDF) [file pcbi.1007189.s008.pdf]

S1 Table. Different scenarios of the 10 migration rate GLMs.

Phylogeographic model	Predictors		Dataset
MASCOT	Time dependent	include sample size	526 HA sequences
MASCOT	Time dependent	exclude sample size	526 HA sequences
MASCOT	Time independent	include sample size	526 HA sequences
MASCOT	Time independent	exclude sample size	526 HA sequences
MASCOT	Time dependent	include sample size	385 HA sequences
MASCOT	Time dependent	exclude sample size	385 HA sequences
DTA	Time independent	include sample size	526 HA sequences
DTA	Time independent	exclude sample size	526 HA sequences
DTA	Time independent	include sample size	385 HA sequences
DTA	Time independent	exclude sample size	385 HA sequences
