# Supplementary material for: Bayesian phylodynamics of avian influenza A virus H9N2 in Asia with time-dependent predictors of migration
Source: PLoS Comput Biol. 2019 Aug 6;15(8):e1007189. doi: 10.1371/journal.pcbi.1007189 (PMC6684064; doi:10.1371/journal.pcbi.1007189)
Supplement: S2 Table — (PDF) [file pcbi.1007189.s009.pdf]

S2 Table. Predictors considered to inform H9N2 migration rates in GLMs.

Predictor	Predictor description	Temporal information	If included in GLMs
poultry trade	The number of live poultry (including chickens, ducks and turkeys) was exchanged between locations by trade, log-transformed, standardised	Yearly	Yes
poultry production origin	The number of live poultry production (including chickens, ducks and turkeys) in each origin location, log-transformed, standardised	Yearly	Yes
poultry production destination	The number of live poultry production (including chickens, ducks and turkeys) in each destination location, log-transformed, standardised	Yearly	Yes
share border	A binary variable describes if the two locations share border on the continent	Constant	Yes
Geographic distance	Great circle distances between geographic centroids of locations, log-transformed, standardised	Constant	Yes
Temperature origin	The mean temperature in origin location, log-transformed, standardised	Yearly	Yes
Temperature destination	The mean temperature in destination location, log-transformed, standardised	Yearly	Yes
Temperature season origin	It describes the variation of monthly temperature in origin location, log-transformed, standardised	Yearly	No
Temperature season destination	It describes the variation of monthly temperature in destination location, log-transformed, standardised	Yearly	No
Rainfall origin	The total rainfall in origin location, log-transformed, standardised	Yearly	Yes
Rainfall destination	The total rainfall in destination location, log-transformed, standardised	Yearly	Yes
Rainfall season origin	It describes the variation of monthly rainfall in origin location, log-transformed, standardised	Yearly	Yes
Rainfall season destination	It describes the variation of monthly rainfall in destination location, log-transformed, standardised	Yearly	Yes
Sample size origin	The number of H9N2 virus isolates with full-length HA genes in the origin region, log-transformed, standardised	Yearly	Dependent
Sample size destination	The number of H9N2 virus isolates with full-length HA genes in the destination region, log-transformed, standardised	Yearly	Dependent
Latitude	The latitude of locations geographic centroids, log-transformed, standardised	Constant	No
