# Supplementary material for: Bayesian phylodynamics of avian influenza A virus H9N2 in Asia with time-dependent predictors of migration
Source: PLoS Comput Biol. 2019 Aug 6;15(8):e1007189. doi: 10.1371/journal.pcbi.1007189 (PMC6684064; doi:10.1371/journal.pcbi.1007189)
Supplement: S3 Table — The predictor with BF over 3 was considered as a suggestive support one. The column names except the first two columns showed the name of different migration rates GLM models in this study. In these columns, value 1 in each cell represents the predictor is a suggestive support one in the corresponding model; 0 represents the predictor is not suggestively supported. The total column represents the total times of each predictor was chosen as suggestive support in the 10 GLMs investigated. (PDF) [file pcbi.1007189.s010.pdf]

S3 Table. Times each predictor was selected with suggestive support in 10 migration rate GLMs. The predictor with BF over 3 was considered as a suggestive support one. The column names except the first two columns showed the name of different migration rates GLM models in this study. In these columns, value 1 in each cell represents the predictor is a suggestive support one in the corresponding model; 0 represents the predictor is not suggestively supported. The total column represents the total times of each predictor was chosen as suggestive support in the 10 GLMs investigated.

predictors	total	mascot time 526.s	mascot time 526.ns	mascot time 385.s	mascot time 385.ns	mascot avg 526.s	mascot avg 526.ns	DTA 526.s	DTA 526.ns	DTA 385.s	DTA 385.ns
Distance	10	1	1	1	1	1	1	1	1	1	1
Rainfall.season.to	9	1	1	1	1	1	1	1	1	0	1
Share.border	8	1	1	1	1	1	1	0	0	1	1
Trade	8	1	1	1	1	0	0	1	1	1	1
Production.from	6	1	1	1	1	1	1	0	0	0	0
Sample.size.from	5	1	0	1	0	1	0	1	0	1	0
Rainfall.season.from	4	1	1	0	0	1	1	0	0	0	0
Temperature.from	3	0	0	1	0	1	1	0	0	0	0
Temperature.to	2	0	1	0	1	0	0	0	0	0	0
Production.to	1	0	1	0	0	0	0	0	0	0	0
Sample.size.to	1	0	0	0	0	1	0	0	0	0	0
Rainfall.from	0	0	0	0	0	0	0	0	0	0	0
Rainfall.to	0	0	0	0	0	0	0	0	0	0	0
