# Supplementary material for: Bayesian phylodynamics of avian influenza A virus H9N2 in Asia with time-dependent predictors of migration
Source: PLoS Comput Biol. 2019 Aug 6;15(8):e1007189. doi: 10.1371/journal.pcbi.1007189 (PMC6684064; doi:10.1371/journal.pcbi.1007189)
Supplement: S5 Table — In the column “Label”, D1 points to sequences exclusively in the data set with 526 HA genes; D2 points to sequences simultaneously in both data sets with 526 and 385 HA genes; G1, G9, and Korea represent the representative sequences; C represents outliers in sequences detected by clock test in TempEst; S represents the removed sequences from phylogeographic inference by down-sampling; L represents the removed sequences from phylogeographic inferences in countries with less than 10 isolates in total; P represents the sequences with partial length. All sequences shown here were used in spatiotemporal analysis of H9N2 virus; Sequences labeled by D1, D2 and the representatives were used in phylogeographic reconstructions. (PDF) [file pcbi.1007189.s012.pdf]

S5 Table. Details about H9N2 HA nucleotide sequences used in our analyses. In the column "Label", D1 points to sequences exclusively in the data set with 526 HA genes; D2 points to sequences simultaneously in both data sets with 526 and 385 HA genes; G1, G9, and Korea represent the representative sequences; C represents outliers in sequences detected by clock test in TempEst; S represents the removed sequences from phylogeographic inference by down-sampling; L represents the removed sequences from phylogeographic inferences in countries with less than 10 isolates in total; P represents the sequences with partial length. All sequences shown here were used in spatiotemporal analysis of H9N2 virus; Sequences labeled by D1, D2 and the representatives were used in phylogeographic reconstructions.

Accession	Location	Date	Host	Label	Database
AF156378	Hong Kong	1997	Quail	G1	NCBI
AF156376	Hong Kong	1997	duck	G9	NCBI
KF188265	Hong Kong	1997	Duck	Korea	NCBI
KF188294	China	1994	Chicken	G9	NCBI
KF188366	Hong Kong	1997	Chicken	G9	NCBI
AF218087	Hong Kong	1976	duck	P	NCBI
KF188393	Hong Kong	1976	duck	D2	NCBI
AF523386	Hong Kong	1976	duck	P	NCBI
AF156382	Hong Kong	1977	duck	P	NCBI
AY206674	Hong Kong	1978	duck	D2	NCBI
AF523384	Hong Kong	1978	duck	P	NCBI
AY206672	Hong Kong	1979	duck	S	NCBI
AY206678	Hong Kong	1979	duck	D2	NCBI
AY206679	Hong Kong	1979	duck	D2	NCBI
AY206680	Hong Kong	1979	duck	D2	NCBI

CY031267	Hong Kong	1979	duck	S	NCBI
CY031275	Hong Kong	1979	duck	D2	NCBI
AF218091	India	1986	duck	P	NCBI
AY206675	Hong Kong	1988	quail	D2	NCBI
AF156381	Hong Kong	1992	Quail	P	NCBI
AF384557	China	1994	chicken	D2	NCBI
AF156379	Hong Kong	1994	Chicken	P	NCBI
AF536689	China	1995	Chicken	P	NCBI
AF203008	South Korea	1996	Chicken	D2	NCBI
AF461526	China	1996	Chicken	S	NCBI
AF461527	China	1996	Chicken	D2	NCBI
GU053194	South Korea	1996	chicken	D2	NCBI
KF188387	South Korea	1996	chicken	D2	NCBI
KF188345	South Korea	1996	chicken	D2	NCBI
DQ064376	China	1996	chicken	D2	NCBI
DQ064377	China	1996	chicken	D2	NCBI
AF508574	China	1996	Quail	P	NCBI
GU053186	South Korea	1996	chicken	D2	NCBI
AF536693	China	1996	Chicken	P	NCBI
AF156384	South Korea	1996	Chicken	P	NCBI
AF218105	Singapore	1997	pekin duck	P	NCBI
AF461530	China	1997	Chicken	D2	NCBI
AY330336	Japan	1997	duck	P	NCBI
AB432938	Hong Kong	1997	duck	D2	NCBI

KY785891	Hong Kong	1997	quail	P	NCBI
DQ064380	China	1997	chicken	D1	NCBI
DQ064373	China	1997	duck	D2	NCBI
AF156375	Hong Kong	1997	Pigeon	P	NCBI
AF508564	China	1997	Chicken	P	NCBI
DQ064362	China	1997	chicken	D2	NCBI
DQ064374	China	1997	duck	D2	NCBI
DQ064360	China	1997	chicken	D2	NCBI
AB049159	Japan	1997	parakeet	D2	NCBI
AF508566	China	1997	Chicken	P	NCBI
AF508569	China	1997	Chicken	P	NCBI
AB256666	Japan	1997	chicken	D2	NCBI
AF536690	China	1997	Chicken	P	NCBI
AF536692	China	1997	Chicken	P	NCBI
AF156374	Hong Kong	1997	Chicken	P	NCBI
KY785896	Hong Kong	1997	quail	S	NCBI
JF916713	China	1997-09-13	Muscovy duck	D1	NCBI
JQ344328	Malaysia	1997-09-24	duck	L	NCBI
AF218106	China	1998	unknown	P	NCBI
AF218108	Malaysia	1998	pekin duck	P	NCBI
AF218109	Iran	1998	chicken	P	NCBI
AF218110	Saudi Arabia	1998	chicken	P	NCBI
AF461510	China	1998	Chicken	D2	NCBI
AF461511	China	1998	chicken	S	NCBI

AF461517	China	1998	Chicken	D2	NCBI
AF461520	China	1998	chicken	D2	NCBI
AY043014	China	1998	Chicken	P	NCBI
AY264870	Iran	1998	chicken	P	NCBI
AY264876	Iran	1998	chicken	P	NCBI
AF508565	China	1998	Chicken	P	NCBI
AY345930	Iran	1998	chicken	P	NCBI
AY345931	Iran	1998	chicken	P	NCBI
AY345932	Iran	1998	chicken	P	NCBI
AY345933	Iran	1998	chicken	P	NCBI
AY623810	China	1998	chicken	P	NCBI
AJ781823	Pakistan	1998	chicken	P	NCBI
EU477241	Iran	1998	chicken	D2	NCBI
FJ190113	China	1998	chicken	S	NCBI
FJ794817	Iran	1998	chicken	D2	NCBI
JQ419725	Iran	1998	chicken	P	NCBI
KU357035	Iran	1998	chicken	S	NCBI
DQ064354	China	1998	chicken	D1	NCBI
EU573939	China	1998	chicken	D2	NCBI
CY081264	Saudi Arabia	1998	chicken	S	NCBI
DQ064379	China	1998	chicken	S	NCBI
DQ064370	China	1998	chicken	D2	NCBI
AB049160	Japan	1998	parakeet	D2	NCBI
DQ997448	China	1998	chicken	P	NCBI

AF536694	China	1998	Chicken	P	NCBI
AF536695	China	1998	Chicken	P	NCBI
AF536696	China	1998	Chicken	P	NCBI
AF536698	China	1998	Chicken	P	NCBI
JF795136	China	1998	chicken	D1	NCBI
JN381626	China	1998-01	chicken	S	NCBI
JQ344326	Malaysia	1998-01-06	duck	L	NCBI
KF800947	Iran	1998-02-02	chicken	D2	NCBI
KF313568	China	1998-03	chicken	S	NCBI
FJ793380	China	1998-04-20	chicken	P	NCBI
JX465626	Iran	1998-06	chicken	D2	NCBI
GQ497118	Iran	1998-10-26	chicken	P	NCBI
GQ497117	Iran	1998-11-15	chicken	S	NCBI
AF186268	Hong Kong	1999	Silkie Chicken	P	NCBI
AF218111	North Korea	1999	Chicken	P	NCBI
AF218112	Iran	1999	chicken	P	NCBI
AF218113	Pakistan	1999	chicken	P	NCBI
AF218114	Pakistan	1999	chicken	P	NCBI
AF218116	Pakistan	1999	chicken	P	NCBI
AF218117	Pakistan	1999	chicken	P	NCBI
AF218119	Saudi Arabia	1999	chicken	P	NCBI
AF218120	Saudi Arabia	1999	chicken	P	NCBI
AY036880	China	1999	chicken	D1	NCBI
AF461509	China	1999	chicken	S	NCBI

AF461512	China	1999	Chicken	S	NCBI
AF461516	China	1999	Chicken	S	NCBI
AF461521	China	1999	Chicken	S	NCBI
AF461522	China	1999	chicken	S	NCBI
AF461528	China	1999	Chicken	D2	NCBI
AY206676	Hong Kong	1999	guinea fowl	D2	NCBI
AY206677	Hong Kong	1999	chicken	D2	NCBI
AY198316	Iran	1999	chicken	P	NCBI
AY198317	Iran	1999	chicken	P	NCBI
AY264875	Iran	1999	chicken	P	NCBI
AY345936	Iran	1999	chicken	P	NCBI
AY330334	Japan	1999	duck	P	NCBI
AJ781824	United Arab Emirates	1999	chicken	P	NCBI
DQ227352	China	1999	chicken	S	NCBI
EU477247	Iran	1999	chicken	D2	NCBI
FJ190148	China	1999	chicken	D2	NCBI
GQ477293	China	1999	chicken	P	NCBI
AF222607	Hong Kong	1999	Pigeon	P	NCBI
AF508556	Pakistan	1999	chicken	P	NCBI
AF508571	China	1999	Chicken	P	NCBI
DQ064364	China	1999	chicken	D1	NCBI
AF222611	Hong Kong	1999	Chicken	P	NCBI
AF222612	Hong Kong	1999	Chicken	P	NCBI
AF222613	Hong Kong	1999	Silkie Chicken	P	NCBI

AF222608	Hong Kong	1999	Chicken	P	NCBI
AF222610	Hong Kong	1999	pheasant	P	NCBI
DQ064363	China	1999	chicken	D1	NCBI
DQ064375	China	1999	chicken	D2	NCBI
AF222606	Hong Kong	1999	Quail	P	NCBI
AF508568	China	1999	Chicken	P	NCBI
DQ997505	China	1999	chicken	P	NCBI
EF070733	China	1999	chicken	S	NCBI
EU081864	China	1999	chicken	D1	NCBI
AF536691	China	1999	chicken	P	NCBI
AF186269	Hong Kong	1999	Chicken	P	NCBI
AF536697	China	1999	chicken	P	NCBI
HQ117886	China	1999	chicken	S	NCBI
AF186267	Hong Kong	1999	Quail	P	NCBI
DQ681221	China	1999-01	duck	S	NCBI
CY077084	China	1999-02-21	chicken	D2	NCBI
FJ793436	China	1999-03-10	chicken	P	NCBI
HM773438	China	1999-04	chicken	D2	NCBI
GQ497120	Iran	1999-08-29	chicken	S	NCBI
GQ497119	Iran	1999-10-08	chicken	S	NCBI
FJ190112	China	1999-11	chicken	S	NCBI
FJ190126	China	1999-11	chicken	D1	NCBI
HM773437	China	1999-11	chicken	S	NCBI
AF461515	China	2000	chicken	S	NCBI

AF461518	China	2000	chicken	S	NCBI
AF461519	China	2000	Chicken	S	NCBI
AF461523	China	2000	chicken	S	NCBI
AF461529	China	2000	Chicken	D2	NCBI
AF461531	China	2000	chicken	S	NCBI
AY198315	Iran	2000	chicken	P	NCBI
AY198319	Iran	2000	chicken	P	NCBI
AY198320	Iran	2000	chicken	P	NCBI
AY198321	Iran	2000	chicken	P	NCBI
AY264872	Iran	2000	chicken	P	NCBI
AY264874	Iran	2000	chicken	P	NCBI
AF523375	China	2000	Duck	P	NCBI
AY345926	Iran	2000	chicken	P	NCBI
AY345928	Iran	2000	chicken	P	NCBI
AY345929	Iran	2000	chicken	P	NCBI
AY345934	Iran	2000	chicken	P	NCBI
AY345935	Iran	2000	chicken	P	NCBI
AY345940	Iran	2000	chicken	P	NCBI
AJ536330	Iran	2000	chicken	P	NCBI
AJ536332	Iran	2000	chicken	P	NCBI
AY513715	China	2000	chicken	S	NCBI
AY594194	China	2000	chicken	P	NCBI
AY768552	South Korea	2000	chicken	P	NCBI
AY768553	South Korea	2000	chicken	P	NCBI

AY768554	South Korea	2000	chicken	P	NCBI
AY768555	South Korea	2000	chicken	P	NCBI
AY768556	South Korea	2000	chicken	P	NCBI
AY768557	South Korea	2000	chicken	P	NCBI
AY768558	South Korea	2000	chicken	P	NCBI
AJ781825	Iran	2000	chicken	P	NCBI
DQ104472	Israel	2000	chicken	P	NCBI
DQ473608	South Korea	2000	chicken	P	NCBI
DQ473609	South Korea	2000	chicken	P	NCBI
DQ473610	South Korea	2000	chicken	P	NCBI
DQ473611	South Korea	2000	chicken	P	NCBI
DQ473612	South Korea	2000	chicken	P	NCBI
DQ473613	South Korea	2000	chicken	P	NCBI
EU477248	Iran	2000	chicken	D2	NCBI
EU477249	Iran	2000	chicken	D2	NCBI
FJ357839	Iran	2000	chicken	P	NCBI
KF188389	China	2000	Duck	D1	NCBI
DQ064356	China	2000	chicken	D1	NCBI
AF523377	China	2000	Duck	P	NCBI
AF523382	China	2000	Duck	P	NCBI
AY180454	China	2000	Duck	P	NCBI
AF523378	China	2000	Duck	P	NCBI
AF523380	China	2000	Duck	P	NCBI
AY180447	China	2000	Chicken	P	NCBI

DQ064358	China	2000	chicken	S	NCBI
AF523374	China	2000	Duck	P	NCBI
AF523383	China	2000	Duck	P	NCBI
AY180451	China	2000	Duck	P	NCBI
DQ064355	China	2000	chicken	S	NCBI
DQ064365	China	2000	chicken	S	NCBI
DQ064372	China	2000	chicken	D2	NCBI
AF508567	China	2000	Chicken	P	NCBI
AF523372	China	2000	Duck	P	NCBI
AF523373	China	2000	Duck	P	NCBI
DQ064366	China	2000	chicken	S	NCBI
DQ064368	China	2000	chicken	D2	NCBI
EF154909	China	2000	quail	P	NCBI
EF154910	China	2000	quail	P	NCBI
EF154912	China	2000	quail	P	NCBI
EF063512	United Arab Emirates	2000	quail	D2	NCBI
EF154907	China	2000	quail	P	NCBI
EF063511	United Arab Emirates	2000	quail	D2	NCBI
EF620900	South Korea	2000	unknown	D2	NCBI
EF154908	China	2000	quail	P	NCBI
EF063510	United Arab Emirates	2000	quail	D2	NCBI
EF154911	China	2000	quail	P	NCBI
EF154913	China	2000	quail	P	NCBI
CY024120	China	2000	partridge	P	NCBI

CY024296	China	2000	partridge	P	NCBI
CY024568	China	2000	chicken	P	NCBI
CY024608	China	2000	chicken	P	NCBI
CY024624	China	2000	chicken	P	NCBI
EU086237	China	2000	chicken	S	NCBI
CY023976	China	2000	Guinea fowl	P	NCBI
CY024208	China	2000	partridge	P	NCBI
CY024616	China	2000	Silkie Chicken	P	NCBI
CY023091	China	2000	partridge	P	NCBI
CY024576	China	2000	chicken	P	NCBI
CY024584	China	2000	chicken	P	NCBI
CY024592	China	2000	chicken	P	NCBI
CY024600	China	2000	chicken	P	NCBI
CY024384	China	2000	partridge	P	NCBI
EF492221	Israel	2000	chicken	D2	NCBI
FJ190124	China	2000-02	chicken	S	NCBI
CY006018	China	2000-02-17	quail	S	NCBI
CY006021	China	2000-02-17	wild duck	S	NCBI
GQ497122	Iran	2000-07-19	chicken	S	NCBI
GQ497121	Iran	2000-09-14	chicken	S	NCBI
DQ485216	China	2000-11-13	chicken	D2	NCBI
DQ485224	China	2000-11-20	chicken	S	NCBI
HM773441	China	2000-12	chicken	S	NCBI
AF461524	China	2001	Chicken	S	NCBI

AF461525	China	2001	Chicken	S	NCBI
AY083840	China	2001	Chicken	D2	NCBI
AY083841	China	2001	Chicken	S	NCBI
AY198313	Iran	2001	chicken	P	NCBI
AY198318	Iran	2001	chicken	P	NCBI
AY264873	Iran	2001	chicken	P	NCBI
AF523379	China	2001	Duck	P	NCBI
AY345925	Iran	2001	chicken	P	NCBI
AY345927	Iran	2001	chicken	P	NCBI
AY345938	Iran	2001	chicken	P	NCBI
AY345939	Iran	2001	chicken	P	NCBI
AJ536331	Iran	2001	chicken	P	NCBI
AY548499	Israel	2001	turkey	P	NCBI
AY548500	Israel	2001	chicken	P	NCBI
AY548501	Israel	2001	chicken	P	NCBI
AY594195	China	2001	chicken	P	NCBI
AY768559	South Korea	2001	chicken	P	NCBI
AJ781827	China	2001	chicken	P	NCBI
DQ104465	Israel	2001	chicken	P	NCBI
DQ104469	Israel	2001	geese	P	NCBI
DQ473614	South Korea	2001	chicken	P	NCBI
DQ681207	China	2001	duck	S	NCBI
DQ681216	China	2001	duck	D2	NCBI
FJ190131	China	2001	chicken	S	NCBI

FJ190132	China	2001	chicken	D2	NCBI
GQ477290	China	2001	chicken	P	NCBI
JX094859	South Korea	2001	chicken	D2	NCBI
KF188377	China	2001	duck	S	NCBI
EU253561	South Korea	2001	chicken	D2	NCBI
DQ064371	China	2001	chicken	D1	NCBI
AB256746	Japan	2001	chicken	D1	NCBI
AF523381	China	2001	wild duck	P	NCBI
DQ064359	China	2001	chicken	S	NCBI
DQ064361	China	2001	chicken	D1	NCBI
DQ064378	China	2001	chicken	S	NCBI
AF523376	China	2001	Duck	P	NCBI
AY180444	China	2001	Chicken	P	NCBI
AB256682	Japan	2001	chicken	D2	NCBI
AB256730	Japan	2001	chicken	D2	NCBI
AB256722	Japan	2001	chicken	D2	NCBI
DQ064367	China	2001	chicken	S	NCBI
AB256674	Japan	2001	chicken	D1	NCBI
EF154919	China	2001	quail	P	NCBI
EF154923	China	2001	quail	P	NCBI
EF154927	China	2001	quail	P	NCBI
DQ997460	China	2001	chicken	P	NCBI
EF154917	China	2001	quail	P	NCBI
EF154922	China	2001	quail	P	NCBI

EF154925	China	2001	quail	P	NCBI
EF154926	China	2001	quail	P	NCBI
EF154928	China	2001	quail	P	NCBI
DQ997474	China	2001	chicken	P	NCBI
EF154916	China	2001	quail	P	NCBI
EF154918	China	2001	quail	P	NCBI
EF154920	China	2001	quail	P	NCBI
EF154914	China	2001	quail	P	NCBI
EF154924	China	2001	quail	P	NCBI
EF154915	China	2001	quail	P	NCBI
AB256738	Japan	2001	chicken	D2	NCBI
AB256690	Japan	2001	chicken	D2	NCBI
AB256698	Japan	2001	chicken	D1	NCBI
DQ997465	China	2001	chicken	P	NCBI
EF154921	China	2001	quail	P	NCBI
EF063513	United Arab Emirates	2001	chicken	D2	NCBI
CY023096	China	2001	partridge	P	NCBI
CY023272	China	2001	partridge	P	NCBI
CY024688	China	2001	chicken	P	NCBI
CY024704	China	2001	chicken	P	NCBI
CY023928	China	2001	duck	P	NCBI
CY023184	China	2001	partridge	P	NCBI
CY023360	China	2001	partridge	P	NCBI
CY024632	China	2001	chicken	P	NCBI

CY024696	China	2001	chicken	P	NCBI
CY024712	China	2001	chicken	P	NCBI
CY023936	China	2001	duck	P	NCBI
CY024720	China	2001	chicken	P	NCBI
CY024728	China	2001	chicken	P	NCBI
CY023104	China	2001	chicken	P	NCBI
EU753298	China	2001	chicken	S	NCBI
CY024472	China	2001	partridge	P	NCBI
CY024560	China	2001	Guinea fowl	P	NCBI
CY024648	China	2001	partridge	P	NCBI
CY024640	China	2001	chicken	P	NCBI
CY024656	China	2001	chicken	P	NCBI
CY024664	China	2001	chicken	P	NCBI
CY024672	China	2001	chicken	P	NCBI
CY024680	China	2001	Silkie Chicken	P	NCBI
CY023912	China	2001	duck	P	NCBI
CY023920	China	2001	duck	P	NCBI
EU753306	China	2001	chicken	D1	NCBI
EU753314	China	2001	chicken	S	NCBI
EU753322	China	2001	chicken	S	NCBI
EU753330	China	2001	chicken	S	NCBI
EF492233	Israel	2001	turkey	D2	NCBI
EU573938	China	2001	chicken	S	NCBI
CY073800	Malaysia	2001	duck	L	NCBI

FJ190114	China	2001-01	chicken	S	NCBI
FJ190129	China	2001-02	chicken	D1	NCBI
KF844248	China	2001-02	chicken	S	NCBI
FJ190111	China	2001-03	chicken	S	NCBI
KF313566	China	2001-04	chicken	S	NCBI
GQ497123	Iran	2001-04-09	chicken	D2	NCBI
FJ793396	China	2001-04-10	chicken	P	NCBI
CY006025	China	2001-04-27	duck	S	NCBI
FJ190115	China	2001-05	chicken	S	NCBI
KF746827	China	2001-07-18	chicken	D2	NCBI
GQ497124	Iran	2001-07-22	chicken	S	NCBI
FJ190125	China	2001-10	chicken	D1	NCBI
FJ190127	China	2001-10	chicken	S	NCBI
FJ190150	China	2001-10	chicken	S	NCBI
AY738454	Israel	2001-12-07	chicken	D2	NCBI
AB634252	China	2001-12-09	chicken	S	NCBI
AY281745	China	2002	chicken	S	NCBI
AY264871	Iran	2002	chicken	P	NCBI
AY364228	China	2002	chicken	S	NCBI
AY548510	Israel	2002	turkey	P	NCBI
AY548512	Israel	2002	turkey	P	NCBI
AY548513	Israel	2002	turkey	P	NCBI
AY594196	China	2002	chicken	P	NCBI
DQ104448	Israel	2002	turkey	P	NCBI

DQ104449	Israel	2002	turkey	P	NCBI
DQ104450	Israel	2002	turkey	P	NCBI
DQ104451	Israel	2002	turkey	P	NCBI
DQ104452	Israel	2002	turkey	P	NCBI
DQ104462	Israel	2002	turkey	P	NCBI
DQ104471	Israel	2002	turkey	P	NCBI
EU304455	China	2002	chicken	P	NCBI
EU477246	Iran	2002	chicken	D2	NCBI
GQ477291	China	2002	chicken	P	NCBI
GQ477294	China	2002	chicken	P	NCBI
DQ064357	China	2002	chicken	D2	NCBI
AB256714	Japan	2002	chicken	D2	NCBI
DQ064369	China	2002	chicken	D1	NCBI
AB256706	Japan	2002	chicken	D2	NCBI
DQ997497	China	2002	chicken	P	NCBI
DQ997481	China	2002	chicken	P	NCBI
DQ997490	China	2002	chicken	P	NCBI
EF154931	China	2002	quail	P	NCBI
DQ997187	China	2002	chicken	P	NCBI
EF154936	China	2002	quail	P	NCBI
EF154932	China	2002	quail	P	NCBI
EF154934	China	2002	quail	P	NCBI
EF154937	China	2002	quail	P	NCBI
EF154938	China	2002	quail	P	NCBI

EF154939	China	2002	quail	P	NCBI
EF063515	United Arab Emirates	2002	chicken	P	NCBI
DQ997451	China	2002	duck	P	NCBI
EF154935	China	2002	quail	P	NCBI
EF154930	China	2002	quail	P	NCBI
CY023888	China	2002	partridge	P	NCBI
CY024048	China	2002	partridge	P	NCBI
CY023128	China	2002	chicken	P	NCBI
CY023144	China	2002	chicken	P	NCBI
CY023864	China	2002	chicken	P	NCBI
CY023944	China	2002	duck	P	NCBI
CY023800	China	2002	chukkar	P	NCBI
CY023984	China	2002	chukkar	P	NCBI
CY024056	China	2002	partridge	P	NCBI
CY023120	China	2002	chicken	P	NCBI
CY023136	China	2002	chicken	P	NCBI
CY023856	China	2002	chicken	P	NCBI
CY023952	China	2002	duck	P	NCBI
CY023448	China	2002	partridge	P	NCBI
CY023536	China	2002	chukkar	P	NCBI
CY023624	China	2002	partridge	P	NCBI
CY023712	China	2002	chukkar	P	NCBI
CY023112	China	2002	chicken	P	NCBI
CY023848	China	2002	chicken	P	NCBI

CY023960	China	2002	duck	P	NCBI
CY023152	China	2002	chicken	P	NCBI
CY023160	China	2002	chicken	P	NCBI
CY023768	China	2002	chicken	P	NCBI
GQ335466	China	2002	chicken	P	NCBI
EF492237	Israel	2002	turkey	D2	NCBI
EF492229	Israel	2002	turkey	D2	NCBI
FJ384751	China	2002	chicken	S	NCBI
GU050554	Saudi Arabia	2002	chicken	D2	NCBI
FJ190123	China	2002-01	chicken	D2	NCBI
FJ190122	China	2002-02	chicken	S	NCBI
FJ190128	China	2002-02	chicken	S	NCBI
FJ190130	China	2002-02	chicken	S	NCBI
FJ793324	China	2002-02-10	chicken	P	NCBI
EF462494	Israel	2002-02-17	chicken	P	NCBI
KF313565	China	2002-03	duck	S	NCBI
KJ162124	China	2002-03-20	chicken	S	NCBI
JN381627	China	2002-04	chicken	D2	NCBI
JQ710462	China	2002-05	chicken	D2	NCBI
GU471884	China	2002-05-10	chicken	D1	NCBI
EF462495	Israel	2002-06-15	turkey	P	NCBI
KF374942	Iran	2002-07-31	chicken	P	NCBI
GQ497125	Iran	2002-08-13	chicken	P	NCBI
FJ793364	China	2002-08-15	chicken	P	NCBI

FJ793444	China	2002-10-20	duck	P	NCBI
GU471885	China	2002-11-04	chicken	D1	NCBI
FJ793300	China	2002-11-10	duck	P	NCBI
GU471882	China	2002-12-02	chicken	D2	NCBI
GQ497126	Iran	2002-12-05	chicken	P	NCBI
KY785737	South Korea	2002-12-07	chicken	S	NCBI
KY785745	South Korea	2002-12-07	chicken	P	NCBI
FJ793316	China	2002-12-20	chicken	P	NCBI
AY336597	India	2003	chicken	P	NCBI
AY435039	India	2003	chicken	P	NCBI
AY435040	India	2003	chicken	P	NCBI
AY548508	Israel	2003	chicken	P	NCBI
AY548509	Israel	2003	chicken	P	NCBI
AY664677	Hong Kong	2003	chicken	P	NCBI
DQ108907	Israel	2003	chicken	P	NCBI
DQ108915	Israel	2003	chicken	P	NCBI
DQ104456	Israel	2003	turkey	P	NCBI
DQ104457	Israel	2003	chicken	P	NCBI
DQ104475	Israel	2003	turkey	P	NCBI
DQ104476	Israel	2003	chicken	P	NCBI
DQ104477	Israel	2003	chicken	P	NCBI
DQ104479	Israel	2003	chicken	P	NCBI
DQ104481	Israel	2003	chicken	P	NCBI
DQ104483	Israel	2003	chicken	P	NCBI

DQ104485	Israel	2003	turkey	P	NCBI
DQ223544	India	2003	chicken	P	NCBI
DQ681203	China	2003	duck	D1	NCBI
DQ922904	Iran	2003	chicken	D2	NCBI
EF063725	Iran	2003	chicken	D2	NCBI
EF063726	Iran	2003	chicken	D2	NCBI
EF063727	Iran	2003	chicken	D2	NCBI
GQ477292	China	2003	chicken	P	NCBI
AB538969	Mongolia	2003	duck	P	NCBI
HM008896	China	2003	chicken	D1	NCBI
HM008897	China	2003	chicken	D2	NCBI
HM008898	China	2003	chicken	S	NCBI
HM008899	China	2003	chicken	D2	NCBI
FN600115	Iran	2003	garganey	D2	NCBI
JX273542	India	2003	chicken	D2	NCBI
JX273553	Pakistan	2003	chicken	D2	NCBI
DQ226108	Hong Kong	2003	Gf	P	NCBI
DQ226107	Hong Kong	2003	pheasant	P	NCBI
AY862602	South Korea	2003	duck	P	NCBI
DQ390215	Jordan	2003	chicken	P	NCBI
EF154949	China	2003	quail	P	NCBI
EF154942	China	2003	quail	P	NCBI
EF154950	China	2003	quail	P	NCBI
EF154956	China	2003	quail	P	NCBI

EF154943	China	2003	quail	P	NCBI
EF154951	China	2003	quail	P	NCBI
EF154953	China	2003	quail	P	NCBI
EF063516	United Arab Emirates	2003	chicken	P	NCBI
EF154940	China	2003	quail	P	NCBI
EF154948	China	2003	quail	P	NCBI
EF154952	China	2003	quail	P	NCBI
EF154944	China	2003	quail	P	NCBI
EF154946	China	2003	quail	P	NCBI
EF154947	China	2003	quail	P	NCBI
EF154954	China	2003	quail	P	NCBI
EF154955	China	2003	quail	P	NCBI
CY024104	China	2003	partridge	P	NCBI
CY024128	China	2003	pheasant	P	NCBI
CY024184	China	2003	chukkar	P	NCBI
CY023192	China	2003	chicken	P	NCBI
CY023208	China	2003	chicken	P	NCBI
CY023880	China	2003	chicken	P	NCBI
CY024096	China	2003	partridge	P	NCBI
CY024112	China	2003	pheasant	P	NCBI
CY024176	China	2003	pheasant	P	NCBI
CY024192	China	2003	pheasant	P	NCBI
CY023200	China	2003	chicken	P	NCBI
CY023216	China	2003	chicken	P	NCBI

CY023872	China	2003	chicken	P	NCBI
CY024008	China	2003	duck	P	NCBI
CY024136	China	2003	chukkar	P	NCBI
CY024144	China	2003	partridge	P	NCBI
CY024152	China	2003	partridge	P	NCBI
CY024160	China	2003	pheasant	P	NCBI
CY023224	China	2003	chicken	P	NCBI
CY023232	China	2003	chicken	P	NCBI
CY023240	China	2003	chicken	P	NCBI
CY023248	China	2003	Silkie Chicken	P	NCBI
CY023256	China	2003	chicken	P	NCBI
CY023968	China	2003	duck	P	NCBI
CY023992	China	2003	duck	P	NCBI
CY024000	China	2003	duck	P	NCBI
CY024064	China	2003	pheasant	P	NCBI
CY024072	China	2003	pheasant	P	NCBI
CY024080	China	2003	partridge	P	NCBI
CY024088	China	2003	chukkar	P	NCBI
CY024200	China	2003	chukkar	P	NCBI
CY024216	China	2003	pheasant	P	NCBI
CY024224	China	2003	partridge	P	NCBI
CY023168	China	2003	chicken	P	NCBI
CY023176	China	2003	chicken	P	NCBI
EF492240	Israel	2003	chicken	D2	NCBI

EF492239	Israel	2003	chicken	D2	NCBI
EF492238	Israel	2003	turkey	D2	NCBI
DQ226115	Hong Kong	2003	chicken	P	NCBI
DQ226109	Hong Kong	2003	chicken	P	NCBI
DQ226112	Hong Kong	2003	chicken	P	NCBI
DQ226113	Hong Kong	2003	chicken	P	NCBI
DQ226111	Hong Kong	2003	Silkie Chicken	P	NCBI
DQ226114	Hong Kong	2003	Silkie Chicken	P	NCBI
AY862599	South Korea	2003	chicken	D2	NCBI
AY664666	Hong Kong	2003	guineafowl	S	NCBI
AY664661	Hong Kong	2003	chicken	D1	NCBI
AY664662	Hong Kong	2003	chicken	D2	NCBI
AY664660	Hong Kong	2003	chicken	S	NCBI
AY664663	Hong Kong	2003	chicken	S	NCBI
AY664664	Hong Kong	2003	chicken	D2	NCBI
AY664665	Hong Kong	2003	chicken	D1	NCBI
AY664676	Hong Kong	2003	chicken	P	NCBI
AY664668	Hong Kong	2003	chicken	D1	NCBI
AY664669	Hong Kong	2003	chicken	D2	NCBI
AY664675	Hong Kong	2003	chicken	D1	NCBI
AY664678	Hong Kong	2003	chicken	P	NCBI
AY790313	South Korea	2003	chicken	D2	NCBI
AY862601	South Korea	2003	chicken	P	NCBI
AY862604	South Korea	2003	chicken	P	NCBI

AY862605	South Korea	2003	chicken	P	NCBI
AY862606	South Korea	2003	chicken	D2	NCBI
AY862600	South Korea	2003	chicken	P	NCBI
AY862603	South Korea	2003	dove	P	NCBI
AY664674	Hong Kong	2003	guineafowl	S	NCBI
AY664673	Hong Kong	2003	pheasant	D2	NCBI
AY664672	Hong Kong	2003	pigeon	P	NCBI
AY862598	South Korea	2003	Silkie Chicken	P	NCBI
EF462497	Israel	2003-01-05	turkey	P	NCBI
FJ190117	China	2003-02	chicken	D2	NCBI
EF462498	Israel	2003-02-16	turkey	P	NCBI
GU471896	China	2003-02-17	chicken	S	NCBI
GU471886	China	2003-03-05	chicken	S	NCBI
GU471892	China	2003-03-05	chicken	D2	NCBI
MF673351	Jordan	2003-03-05	chicken	S	NCBI
MF673375	Jordan	2003-03-08	chicken	S	NCBI
FJ793412	China	2003-03-20	chicken	P	NCBI
FJ793332	China	2003-03-20	chicken	P	NCBI
JF916721	China	2003-04-01	duck	S	NCBI
EF462501	Israel	2003-04-02	turkey	P	NCBI
GQ497128	Iran	2003-04-14	chicken	P	NCBI
CY068643	India	2003-04-14	chicken	D2	NCBI
EU665420	India	2003-04-24	chicken	D2	NCBI
EU665421	India	2003-04-25	chicken	D2	NCBI

AY738455	Israel	2003-05-05	chicken	D2	NCBI
GU471888	China	2003-05-06	chicken	S	NCBI
GU471893	China	2003-05-06	chicken	D1	NCBI
DQ108921	Israel	2003-05-12	chicken	P	NCBI
DQ108906	Israel	2003-05-26	chicken	P	NCBI
KX349955	China	2003-06	duck	S	NCBI
DQ108908	Israel	2003-07-10	chicken	P	NCBI
DQ108910	Israel	2003-07-14	chicken	P	NCBI
GQ497127	Iran	2003-07-17	chicken	S	NCBI
AB634251	China	2003-10-03	chicken	D1	NCBI
AY738456	Israel	2003-10-30	ostrich	D2	NCBI
MF673367	Jordan	2003-11-18	chicken	S	NCBI
MF673383	Jordan	2003-11-18	chicken	S	NCBI
MF673343	Jordan	2003-11-18	chicken	S	NCBI
KF313560	China	2003-12	chicken	S	NCBI
DQ104480	Israel	2003-12-01	chicken	P	NCBI
KP865930	China	2003-12-02	chicken	D1	NCBI
KP865931	China	2003-12-02	chicken	D2	NCBI
DQ104474	Israel	2003-12-04	chicken	P	NCBI
DQ104482	Israel	2003-12-08	chicken	P	NCBI
MF673319	Jordan	2003-12-11	chicken	S	NCBI
MF673359	Jordan	2003-12-11	chicken	S	NCBI
DQ104484	Israel	2003-12-22	chicken	P	NCBI
DQ108911	Israel	2003-12-24	chicken	P	NCBI

DQ108913	Israel	2003-12-28	turkey	P	NCBI
DQ108914	Israel	2003-12-31	chicken	P	NCBI
AY548503	Israel	2004	chicken	P	NCBI
AY548505	Israel	2004	turkey	P	NCBI
AY790314	South Korea	2004	chicken	P	NCBI
AY790315	South Korea	2004	chicken	P	NCBI
AY937403	China	2004	goose	P	NCBI
AY937404	China	2004	goose	P	NCBI
DQ108905	Israel	2004	chicken	P	NCBI
DQ108912	Israel	2004	chicken	P	NCBI
DQ108918	Israel	2004	turkey	P	NCBI
DQ108923	Israel	2004	chicken	P	NCBI
DQ108924	Israel	2004	turkey	P	NCBI
DQ108925	Israel	2004	chicken	P	NCBI
DQ108926	Israel	2004	turkey	P	NCBI
DQ108927	Israel	2004	chicken	P	NCBI
DQ108928	Israel	2004	chicken	P	NCBI
DQ108929	Israel	2004	chicken	P	NCBI
DQ108930	Israel	2004	chicken	P	NCBI
DQ108932	Israel	2004	turkey	P	NCBI
DQ225271	India	2004	chicken	P	NCBI
DQ464352	South Korea	2004	chicken	D2	NCBI
EF063728	Iran	2004	chicken	D2	NCBI
EF063729	Iran	2004	chicken	D2	NCBI

EF063730	Iran	2004	chicken	D2	NCBI
EF063731	Iran	2004	chicken	D2	NCBI
EF492241	Israel	2004	turkey	D1	NCBI
JX273537	Jordan	2004	unknown	L	NCBI
JX273544	Israel	2004	chicken	D2	NCBI
JX273545	Kuwait	2004	chicken	S	NCBI
JX273546	Lebanon	2004	chicken	L	NCBI
KF188399	Pakistan	2004	chicken	D2	NCBI
KF188395	South Korea	2004	chicken	D2	NCBI
KF188309	South Korea	2004	chicken	D2	NCBI
DQ299845	South Korea	2004	chicken	P	NCBI
DQ299837	South Korea	2004	chicken	P	NCBI
DQ299853	South Korea	2004	chicken	P	NCBI
DQ465400	China	2004	duck	D1	NCBI
EF154957	China	2004	quail	P	NCBI
EF154959	China	2004	quail	P	NCBI
EF154960	China	2004	quail	P	NCBI
EF154962	China	2004	quail	P	NCBI
EF154963	China	2004	quail	P	NCBI
EF154967	China	2004	quail	P	NCBI
EF154961	China	2004	quail	P	NCBI
EF154958	China	2004	quail	P	NCBI
EF154964	China	2004	quail	P	NCBI
EF154965	China	2004	quail	P	NCBI

EF154966	China	2004	quail	P	NCBI
CY024248	China	2004	chukkar	P	NCBI
CY024264	China	2004	partridge	P	NCBI
CY024328	China	2004	partridge	P	NCBI
CY024344	China	2004	Guinea fowl	P	NCBI
CY024392	China	2004	partridge	P	NCBI
CY024464	China	2004	pheasant	P	NCBI
CY023264	China	2004	chicken	P	NCBI
CY023288	China	2004	Silkie Chicken	P	NCBI
CY023344	China	2004	chicken	P	NCBI
CY023368	China	2004	Silkie Chicken	P	NCBI
CY023408	China	2004	chicken	P	NCBI
CY023704	China	2004	chicken	P	NCBI
CY024016	China	2004	duck	P	NCBI
CY024032	China	2004	duck	P	NCBI
CY024240	China	2004	pheasant	P	NCBI
CY024256	China	2004	chukkar	P	NCBI
CY024272	China	2004	pheasant	P	NCBI
CY024320	China	2004	pheasant	P	NCBI
CY024336	China	2004	pheasant	P	NCBI
CY024400	China	2004	chukkar	P	NCBI
CY024416	China	2004	Guinea fowl	P	NCBI
CY024480	China	2004	partridge	P	NCBI
CY023280	China	2004	Silkie Chicken	P	NCBI

CY023296	China	2004	chicken	P	NCBI
CY023336	China	2004	chicken	P	NCBI
CY023352	China	2004	chicken	P	NCBI
CY024024	China	2004	duck	P	NCBI
CY024280	China	2004	partridge	P	NCBI
CY024288	China	2004	chukkar	P	NCBI
CY024304	China	2004	partridge	P	NCBI
CY024312	China	2004	chukkar	P	NCBI
CY024432	China	2004	partridge	P	NCBI
CY024440	China	2004	pheasant	P	NCBI
CY024448	China	2004	partridge	P	NCBI
CY024456	China	2004	pheasant	P	NCBI
CY023376	China	2004	chicken	P	NCBI
CY023384	China	2004	chicken	P	NCBI
CY023392	China	2004	chicken	P	NCBI
CY023400	China	2004	Silkie Chicken	P	NCBI
CY023696	China	2004	chicken	P	NCBI
CY024232	China	2004	partridge	P	NCBI
CY024352	China	2004	chukkar	P	NCBI
CY024360	China	2004	partridge	P	NCBI
CY024368	China	2004	partridge	P	NCBI
CY024376	China	2004	chukkar	P	NCBI
CY023304	China	2004	Silkie Chicken	P	NCBI
CY023312	China	2004	chicken	P	NCBI

CY023320	China	2004	chicken	P	NCBI
CY023328	China	2004	chicken	P	NCBI
CY023896	China	2004	chicken	P	NCBI
CY023904	China	2004	chicken	P	NCBI
EF492242	Israel	2004	chicken	D2	NCBI
EF501983	Israel	2004	chicken	D2	NCBI
EF492243	Israel	2004	chicken	D2	NCBI
EU939150	China	2004	chicken	P	NCBI
EU939151	China	2004	chicken	P	NCBI
DQ108919	Israel	2004-01-09	chicken	P	NCBI
DQ108916	Israel	2004-01-13	turkey	P	NCBI
CY068651	India	2004-02-23	chicken	P	NCBI
EU665422	India	2004-03	chicken	D2	NCBI
KF688983	China	2004-03	chicken	D2	NCBI
JQ639778	China	2004-03-02	chicken	D2	NCBI
EF462505	Israel	2004-03-03	chicken	P	NCBI
FJ793372	China	2004-03-10	chicken	P	NCBI
CY068659	India	2004-04-12	chicken	P	NCBI
KX759089	Pakistan	2004-04-22	chicken	S	NCBI
KF746785	China	2004-04-25	chicken	D2	NCBI
MF673327	Jordan	2004-04-26	chicken	S	NCBI
MF673303	Jordan	2004-04-26	chicken	S	NCBI
DQ108920	Israel	2004-05	turkey	P	NCBI
CY068667	India	2004-05-25	chicken	P	NCBI

KX759091	Pakistan	2004-06-02	chicken	S	NCBI
KF746835	China	2004-07-04	chicken	D2	NCBI
GQ497129	Iran	2004-07-08	chicken	P	NCBI
KF313559	China	2004-09	chicken	S	NCBI
CY068672	India	2004-09-13	chicken	P	NCBI
EU665423	India	2004-10	chicken	D2	NCBI
FJ793292	China	2004-10-15	duck	P	NCBI
MF673311	Jordan	2004-10-28	chicken	S	NCBI
HQ143681	South Korea	2004-11	wild bird	P	NCBI
GQ497130	Iran	2004-11-08	chicken	S	NCBI
FJ793420	China	2004-11-10	chicken	P	NCBI
FJ793356	China	2004-11-20	chicken	P	NCBI
KP865928	China	2004-12-02	chicken	D2	NCBI
EF462507	Israel	2004-12-06	chicken	P	NCBI
FJ793340	China	2004-12-15	chicken	P	NCBI
DQ108922	Israel	2005	turkey	P	NCBI
DQ234277	China	2005	duck	P	NCBI
EF063732	Iran	2005	chicken	D2	NCBI
EF063733	Iran	2005	chicken	D2	NCBI
EF063734	Iran	2005	chicken	D2	NCBI
EF063735	Iran	2005	chicken	D2	NCBI
EU086264	China	2005	unknown	D2	NCBI
EU939152	China	2005	chicken	P	NCBI
JX273556	Saudi Arabia	2005	chicken	D2	NCBI

KF188403	Pakistan	2005	chicken	P	NCBI
KF188371	United Arab Emirates	2005	stone curlew	P	NCBI
KF188359	Pakistan	2005	chicken	D2	NCBI
KF188347	Pakistan	2005	chicken	D2	NCBI
KF188337	United Arab Emirates	2005	stone curlew	D2	NCBI
KF188325	Pakistan	2005	chicken	D2	NCBI
KF188323	China	2005	chicken	D2	NCBI
KF188311	United Arab Emirates	2005	poultry	D2	NCBI
KF188260	United Arab Emirates	2005	white bellied bustard	D2	NCBI
KF188258	United Arab Emirates	2005	white bellied bustard	D2	NCBI
KF188254	United Arab Emirates	2005	quail	P	NCBI
KF188244	United Arab Emirates	2005	white bellied bustard	P	NCBI
KF188236	United Arab Emirates	2005	white bellied bustard	D2	NCBI
EF154975	China	2005	quail	P	NCBI
EF154978	China	2005	quail	P	NCBI
EF154979	China	2005	quail	P	NCBI
EF154969	China	2005	quail	P	NCBI
EF492228	Israel	2005	unknown	D2	NCBI
EF492225	Israel	2005	chicken	D2	NCBI
EF492222	Israel	2005	chicken	D1	NCBI
EF154970	China	2005	quail	P	NCBI
EF154971	China	2005	quail	P	NCBI
EF154973	China	2005	quail	P	NCBI
EF492232	Israel	2005	chicken	D2	NCBI

EF492236	Israel	2005	chicken	D1	NCBI
EF492235	Israel	2005	turkey	D2	NCBI
EF154977	China	2005	quail	P	NCBI
EF154972	China	2005	quail	P	NCBI
EF492227	Israel	2005	chicken	D1	NCBI
EF492234	Israel	2005	chicken	D1	NCBI
EF154974	China	2005	quail	P	NCBI
EF154976	China	2005	quail	P	NCBI
EF154968	China	2005	quail	P	NCBI
EF492226	Israel	2005	turkey	D2	NCBI
EF492230	Israel	2005	turkey	S	NCBI
CY024488	China	2005	pheasant	P	NCBI
CY024544	China	2005	partridge	P	NCBI
CY023424	China	2005	Silkie Chicken	P	NCBI
CY023440	China	2005	Silkie Chicken	P	NCBI
CY023488	China	2005	chicken	P	NCBI
CY023504	China	2005	chicken	P	NCBI
CY023568	China	2005	chicken	P	NCBI
CY023584	China	2005	chicken	P	NCBI
CY023648	China	2005	chicken	P	NCBI
CY023664	China	2005	chicken	P	NCBI
CY023728	China	2005	chicken	P	NCBI
CY023784	China	2005	chicken	P	NCBI
CY023808	China	2005	chicken	P	NCBI

EU086226	China	2005	chicken	S	NCBI
CY024496	China	2005	Guinea fowl	P	NCBI
CY024536	China	2005	pheasant	P	NCBI
CY024552	China	2005	chukkar	P	NCBI
CY023416	China	2005	chicken	P	NCBI
CY023432	China	2005	Silkie Chicken	P	NCBI
CY023496	China	2005	chicken	P	NCBI
CY023512	China	2005	chicken	P	NCBI
CY023560	China	2005	chicken	P	NCBI
CY023576	China	2005	chicken	P	NCBI
CY023640	China	2005	chicken	P	NCBI
CY023656	China	2005	chicken	P	NCBI
CY023720	China	2005	chicken	P	NCBI
CY023736	China	2005	chicken	P	NCBI
CY023776	China	2005	chicken	P	NCBI
CY023792	China	2005	chicken	P	NCBI
EU086246	China	2005	unknown	S	NCBI
EU086245	China	2005	chicken	S	NCBI
CY023520	China	2005	chicken	P	NCBI
CY023528	China	2005	chicken	P	NCBI
CY023544	China	2005	chicken	P	NCBI
CY023552	China	2005	chicken	P	NCBI
CY023672	China	2005	chicken	P	NCBI
CY023680	China	2005	chicken	P	NCBI

CY023688	China	2005	chicken	P	NCBI
CY023816	China	2005	chicken	P	NCBI
CY023824	China	2005	chicken	P	NCBI
CY023832	China	2005	chicken	P	NCBI
CY023840	China	2005	chicken	P	NCBI
EU086234	China	2005	duck	S	NCBI
CY024504	China	2005	pheasant	P	NCBI
CY024528	China	2005	pheasant	P	NCBI
CY023456	China	2005	Silkie Chicken	P	NCBI
CY023464	China	2005	chicken	P	NCBI
CY023472	China	2005	chicken	P	NCBI
CY023480	China	2005	chicken	P	NCBI
CY023592	China	2005	chicken	P	NCBI
CY023600	China	2005	chicken	P	NCBI
CY023608	China	2005	chicken	P	NCBI
CY023616	China	2005	chicken	P	NCBI
CY023744	China	2005	chicken	P	NCBI
CY023752	China	2005	chicken	P	NCBI
CY023760	China	2005	chicken	P	NCBI
CY024040	China	2005	duck	P	NCBI
EU086265	China	2005	unknown	D1	NCBI
EU086266	China	2005	unknown	D2	NCBI
EF492223	Israel	2005	turkey	D1	NCBI
EU573940	China	2005	chicken	D1	NCBI

GQ373069	China	2005	duck	P	NCBI
GQ373068	China	2005	duck	S	NCBI
GQ373083	China	2005	chicken	P	NCBI
KF259061	Hong Kong	2005	chicken	P	NCBI
KF259062	Hong Kong	2005	chicken	P	NCBI
KF259063	Hong Kong	2005	chicken	P	NCBI
KF259064	Hong Kong	2005	chicken	P	NCBI
KF259065	Hong Kong	2005	chicken	P	NCBI
KF259066	Hong Kong	2005	pheasant	P	NCBI
KF259067	Hong Kong	2005	Silkie Chicken	P	NCBI
FJ793284	China	2005-01-10	duck	P	NCBI
FJ793348	China	2005-01-20	chicken	P	NCBI
EF462493	Israel	2005-01-23	turkey	P	NCBI
HM773435	China	2005-02	chicken	S	NCBI
MF673335	Jordan	2005-02-16	chicken	S	NCBI
KF313563	China	2005-03	chicken	S	NCBI
GU121382	China	2005-03	black-billed magpie	S	NCBI
KF768240	China	2005-03-10	duck	S	NCBI
HM773440	China	2005-04	chicken	D1	NCBI
KF746756	China	2005-04-23	chicken	D1	NCBI
HM590762	China	2005-05	black-billed magpie	D2	NCBI
HM590770	China	2005-05	black-billed magpie	S	NCBI
GU471889	China	2005-05-04	chicken	S	NCBI
CY038410	Pakistan	2005-05-18	chicken	D2	NCBI

GU722364	China	2005-09-16	sparrow	D2	NCBI
KF374947	Iran	2005-09-24	chicken	P	NCBI
KF374945	Iran	2005-09-25	chicken	P	NCBI
FJ605501	India	2005-10	watercoot	P	NCBI
KF374943	Iran	2005-10-19	chicken	P	NCBI
KF374944	Iran	2005-10-19	chicken	P	NCBI
KF374946	Iran	2005-10-19	chicken	P	NCBI
GU247901	South Korea	2005-10-28	chicken	P	NCBI
GU722365	China	2005-11-03	sparrow	S	NCBI
GQ497131	Iran	2005-11-08	chicken	S	NCBI
KX759093	Pakistan	2005-11-12	egret	S	NCBI
KX759095	Pakistan	2005-11-12	crow	S	NCBI
KX759097	Pakistan	2005-11-21	coot	S	NCBI
HM773433	China	2005-12	chicken	S	NCBI
HM773436	China	2005-12	chicken	S	NCBI
KY785729	South Korea	2005-12-17	chicken	S	NCBI
GQ497132	Iran	2005-12-21	chicken	P	NCBI
CY038442	Pakistan	2005-12-21	chicken	D2	NCBI
CY038418	Pakistan	2005-12-31	chicken	D1	NCBI
EU157934	China	2006	chicken	D1	NCBI
EU216085	China	2006	chicken	D1	NCBI
EU216088	China	2006	chicken	S	NCBI
AB538967	Japan	2006	duck	P	NCBI
AB538968	Japan	2006	duck	P	NCBI

JX273554	Pakistan	2006	chicken	D2	NCBI
JX273555	Saudi Arabia	2006	chicken	D2	NCBI
JX273559	Saudi Arabia	2006	chicken	D2	NCBI
JX273560	Saudi Arabia	2006	chicken	D2	NCBI
JX273563	China	2006	duck	S	NCBI
KF188375	United Arab Emirates	2006	quail	D2	NCBI
KF188329	United Arab Emirates	2006	houbara	P	NCBI
KF188238	United Arab Emirates	2006	dove	D2	NCBI
EF492224	Israel	2006	chicken	D2	NCBI
EU086303	China	2006	unknown	S	NCBI
EU249369	South Korea	2006	shorebird	P	NCBI
EU086283	China	2006	quail	D1	NCBI
EU086284	China	2006	unknown	S	NCBI
FJ464730	Israel	2006	turkey	D2	NCBI
GQ335471	China	2006	chicken	P	NCBI
GQ335495	China	2006	chicken	P	NCBI
EU573941	China	2006	chicken	D2	NCBI
GU050287	Saudi Arabia	2006	unknown	D2	NCBI
GU050295	Saudi Arabia	2006	unknown	D2	NCBI
GU050279	Saudi Arabia	2006	unknown	D1	NCBI
GQ373075	China	2006	chicken	S	NCBI
GQ373071	China	2006	chicken	P	NCBI
GQ373082	China	2006	chicken	P	NCBI
KF259068	Hong Kong	2006	chicken	P	NCBI

KF259069	Hong Kong	2006	chicken	P	NCBI
KF259070	Hong Kong	2006	chicken	P	NCBI
KF259071	Hong Kong	2006	chicken	P	NCBI
KF259072	Hong Kong	2006	chicken	P	NCBI
KF259073	Hong Kong	2006	chicken	P	NCBI
KF259074	Hong Kong	2006	chicken	P	NCBI
KF259075	Hong Kong	2006	pheasant	P	NCBI
KF259076	Hong Kong	2006	Silkie Chicken	P	NCBI
GU247902	South Korea	2006-01-17	chicken	P	NCBI
KC293797	Oman	2006-01-18	chicken	P	NCBI
KC293798	Oman	2006-01-18	chicken	P	NCBI
JN543596	South Korea	2006-01-19	chicken	P	NCBI
KF768215	China	2006-01-25	duck	S	NCBI
GU722360	China	2006-02-07	chicken	D1	NCBI
GU247914	South Korea	2006-02-10	chicken	P	NCBI
GU247903	South Korea	2006-02-13	chicken	P	NCBI
GU247915	South Korea	2006-02-17	chicken	D2	NCBI
JN540058	Pakistan	2006-02-23	chicken	D2	NCBI
HM370056	India	2006-02-25	chicken	S	NCBI
HM370049	India	2006-02-25	chicken	S	NCBI
JQ710463	China	2006-03	chicken	S	NCBI
GU722362	China	2006-04-23	chicken	S	NCBI
GU247916	South Korea	2006-04-27	chicken	P	NCBI
CY038426	Pakistan	2006-05-02	chicken	D2	NCBI

JN543597	South Korea	2006-05-10	chicken	P	NCBI
JN543598	South Korea	2006-05-23	chicken	P	NCBI
GU247904	South Korea	2006-05-25	chicken	P	NCBI
GU247905	South Korea	2006-05-26	chicken	P	NCBI
GU247906	South Korea	2006-05-29	chicken	P	NCBI
GU471883	China	2006-06-08	chicken	S	NCBI
GQ497134	Iran	2006-06-09	chicken	P	NCBI
GU471895	China	2006-06-10	chicken	S	NCBI
CY038402	Pakistan	2006-06-19	chicken	D2	NCBI
FJ190116	China	2006-06-22	chicken	S	NCBI
GU722366	China	2006-06-27	sparrow	D2	NCBI
FJ190135	China	2006-08-20	chicken	D2	NCBI
KC986294	Bangladesh	2006-09	chicken	S	NCBI
GU722367	China	2006-09-11	sparrow	D2	NCBI
GU722363	China	2006-10-17	chicken	S	NCBI
GQ497133	Iran	2006-11-15	chicken	S	NCBI
GU247907	South Korea	2006-11-22	chicken	P	NCBI
AB753177	Viet Nam	2006-12	Chinese Hwamei	D2	NCBI
KP865927	China	2006-12-02	chicken	S	NCBI
KP865929	China	2006-12-02	chicken	D1	NCBI
GU247908	South Korea	2006-12-08	chicken	P	NCBI
FJ464728	Israel	2006-12-10	chicken	D2	NCBI
FJ464729	Israel	2006-12-13	chicken	D2	NCBI
GU722368	China	2006-12-19	sparrow	D1	NCBI

KM411633	China	2006-12-23	chicken	D2	NCBI
FJ464714	Israel	2006-12-26	chicken	D2	NCBI
JN543599	South Korea	2006-12-27	chicken	P	NCBI
CY038434	Pakistan	2006-12-29	chicken	D2	NCBI
EU216080	China	2007	chicken	D1	NCBI
EU216081	China	2007	chicken	S	NCBI
EU216082	China	2007	chicken	S	NCBI
EU216083	China	2007	chicken	S	NCBI
EU216084	China	2007	chicken	D1	NCBI
EU216086	China	2007	chicken	S	NCBI
EU216087	China	2007	chicken	S	NCBI
EU216089	China	2007	chicken	S	NCBI
EU216090	China	2007	chicken	S	NCBI
EU216091	China	2007	chicken	S	NCBI
EU216092	China	2007	chicken	S	NCBI
EU477242	Iran	2007	chicken	S	NCBI
EU477243	Iran	2007	chicken	D1	NCBI
EU477244	Iran	2007	chicken	S	NCBI
EU477245	Iran	2007	chicken	D2	NCBI
EU477539	Iran	2007	chicken	D2	NCBI
EU644482	China	2007	goose	S	NCBI
EU644485	China	2007	chicken	S	NCBI
EU926626	China	2007	duck	D2	NCBI
EU939145	China	2007	chicken	P	NCBI

HM008887	China	2007	chicken	S	NCBI
