# Supplementary material for: Bayesian phylodynamics of avian influenza A virus H9N2 in Asia with time-dependent predictors of migration
Source: PLoS Comput Biol. 2019 Aug 6;15(8):e1007189. doi: 10.1371/journal.pcbi.1007189 (PMC6684064; doi:10.1371/journal.pcbi.1007189)
Supplement: S4 Table — The predictor with BF over 20 was considered as strong support. Parameters here are the same as in S3 Table. (PDF) [file pcbi.1007189.s011.pdf]

S4 Table. Times each predictor was selected with strong support in 10 migration rate GLMs. The predictor with BF over 20 was considered as strong support. Parameters here are the same as in S3 Table.

predictors	total	mascot time 526.s	mascot time 526.ns	mascot time 385.s	mascot time 385.ns	mascot avg 526.s	mascot avg 526.ns	DTA 526.s	DTA 526.ns	DTA 385.s	DTA 385.ns
Distance	10	1	1	1	1	1	1	1	1	1	1
Trade	8	1	1	1	1	0	0	1	1	1	1
Rainfall.season.to	6	1	1	0	1	1	1	0	1	0	0
Share.border	6	1	1	1	1	1	1	0	0	0	0
Rainfall.season.from	4	1	1	0	0	1	1	0	0	0	0
Production.from	3	1	1	0	1	0	0	0	0	0	0
Sample.size.from	2	1	0	1	0	0	0	0	0	0	0
Temperature.from	2	0	0	0	0	1	1	0	0	0	0
Production.to	0	0	0	0	0	0	0	0	0	0	0
Rainfall.from	0	0	0	0	0	0	0	0	0	0	0
Rainfall.to	0	0	0	0	0	0	0	0	0	0	0
Sample.size.to	0	0	0	0	0	0	0	0	0	0	0
Temperature.to	0	0	0	0	0	0	0	0	0	0	0
